# Dopamine and Calcium Dynamics in the Nucleus Accumbens Core during Food Seeking

**DOI:** 10.1523/ENEURO.0380-25.2026

**Published:** 2026-04-28

**Authors:** Sophia J. Weber, Gillian S. Driscoll, Madelyn M. Beutler, Hayley M. Kuhn, Jonathan G. Westlake, Marina E. Wolf

**Affiliations:** Department of Behavioral Neuroscience, Oregon Health & Science University, Portland, Oregon 97239

**Keywords:** calcium, dopamine, fiber photometry, food, nucleus accumbens, self-administration

## Abstract

Extinction-reinstatement paradigms have been used to study reward-seeking for both food and drug rewards. The nucleus accumbens (NAc) is of particular interest in reinstatement due to its ability to energize motivated behavior. Previous work found that suppression of neuronal activity or dopamine signaling in NAc reduces reinstatement of food-seeking. Here we used fiber photometry and sensor multiplexing (red-shifted dopamine sensor and genetically-encoded calcium indicator) to measure dopamine and calcium in NAc core of male and female rats on each day of an extinction-reinstatement paradigm with food reward to determine how signals vary across task phases. During self-administration training, we detected positive dopamine transients that initially followed lever pressing but moved earlier in time as training progressed. A post-press dopamine decrease also emerged with training. For calcium, a decrease from baseline occurred after the press and became more prominent across training. Both patterns were reduced in the first extinction session, with no deflections from baseline detected in dopamine or calcium traces in the last extinction session. During reinstatement tests primed with either cue or combined food reward and cue presentation, we observed positive calcium and dopamine responses that differed significantly from the signals measured in the last extinction session. While multiplexing has been validated by prior studies, ours is the first to simultaneously record dopamine and calcium during an extinction-reinstatement task. The results provide new information about changing relationships between these signals across task phases, setting the stage for exploring their behavioral significance and mechanisms that may link the two signals.

## Significance Statement

The nucleus accumbens (NAc) is a critical region in reward-seeking behavior with dopamine acting on local neurons to energize behavior. Studies examining reward-seeking behavior have typically assessed activity of NAc neurons and dopamine release separately. However, recent studies have validated sensor multiplexing for simultaneous recording of calcium (which is related to neuronal activation state) and dopamine. Here we used sensor multiplexing in rats to assess calcium and dopamine signals in NAc on each day of food self-administration, extinction training, and reinstatement testing. We found changing relationships between calcium and dopamine signaling across phases of this paradigm, providing new information about their interplay and setting the stage for future studies of mechanisms linking these signals and their behavioral significance.

## Introduction

Learned associations between an environmental cue and rewarded outcome are generally adaptive and help guide motivated behavior. However, the ability of reward-associated cues to elicit behavior can become problematic when these rewards also result in negative consequences, as with addictive drugs. Extinction-reinstatement paradigms have been utilized for decades to interrogate drivers of maladaptive reward-seeking behavior, particularly as an analog for relapse in substance use disorder (Shaham et al., 2003; Venniro et al., 2016). This model has also been used with palatable food reward instead of drug reward to study mechanisms of food seeking (Calu et al., 2014; Scofield et al., 2016). It allows for the examination of neurobiological underpinnings of reward learning across multiple phases: acquisition, extinction, and reinstatement.

The nucleus accumbens (NAc) is an important locus for goal-directed behavior, with its principal neurons (medium spiny neurons or MSN) integrating information from cortical, limbic, and midbrain inputs and sending output to downstream basal ganglia regions (Floresco, 2015). The NAc has long been recognized as a particularly important region in food seeking and consummatory behavior (Kelley, 2004; Alonso-Caraballo et al., 2021). Early studies found that a subset of NAc MSN exhibit a pause in firing during initiation and maintenance of sucrose consumption (Nicola et al., 2004; Roitman et al., 2005; Taha and Fields, 2005, 2006), with a subsequent study showing that electrical stimulation of NAc regions containing this subset of neurons interrupts consumption (Krause et al., 2010). In operant tasks, recordings of NAc neurons also reveal subpopulations of MSN with distinct response patterns. For example, a subset of phasically active MSN exhibits an increase in firing prior to lever pressing for food or water reward, with a separate subset showing inhibition immediately before and after responding (Carelli et al., 2000). Furthermore, activity of NAc MSN regulates food seeking in extinction-reinstatement paradigms. For example, pharmacological inactivation of the NAc core (NAcc), but not shell, impaired cue-induced reinstatement (Floresco et al., 2008).

At the level of neurotransmitters in the NAc, dopamine (DA) transmission is important for reward seeking in food tasks (Volkow et al., 2017; Marinescu and Labouesse, 2024) despite not being necessary for the hedonic value of reward (Berridge and Robinson, 1998). For example, blockade of either DA D1 receptors or DA D2 receptors in the NAcc inhibited food reinstatement (Guy et al., 2011). More recently, a series of studies have established the importance of plasticity of glutamate transmission in the NAc for regulation of food seeking (Ferrario, 2020). These findings suggest that DA and glutamatergic input into the NAcc converge to facilitate food reinstatement, which is supported by evidence for these systems working together in other circumstances (Nicola et al., 2000; Wolf, 2010; Tritsch and Sabatini, 2012; Sippy and Tritsch, 2023; Weber et al., 2024).

One approach to understanding this convergence is to use sensor multiplexing to simultaneously measure DA and calcium in the NAcc. Calcium levels are related to the activation state of NAcc MSN and thus their ability to respond to glutamate (see Discussion). Several studies have validated sensor multiplexing for DA and calcium (Patriarchi et al., 2018; 2020) and other sensor combinations (Dana et al., 2016; Kim et al., 2016; Patriarchi et al., 2020; Xie et al., 2024; Fink et al., 2025), although none of these studies applied sensor multiplexing to an operant model. Here we employed fiber photometry with sensor multiplexing to simultaneously record NAcc DA release and MSN calcium levels on each day of a food extinction-reinstatement task. We found that DA release increased during the learning or self-administration phase and that this peak eventually shifted earlier in time to precede the lever press. This DA signal then declined with extinction and re-emerged with reinstatement. In contrast, MSN calcium levels decreased from baseline after the lever press during self-administration. This decrease disappeared after the first day of extinction, and reinstatement was associated with an increase in MSN calcium.

Our study is the first to measure calcium and DA in the same animal across all phases of an extinction-reinstatement task. Our results add to the literature by revealing time-dependent changes for both DA and calcium, as well as changing relationships between the two signals, across phases of this operant task. A challenging but important next step will be to assess potential causal relationships between DA signaling, calcium changes, and behavior in this paradigm. It will also be interesting in the future to compare these results to those obtained with drugs of abuse.

## Materials and Methods

### Subjects

We used Long–Evans rats (6 male and 5 female) obtained from Charles River. They were ∼9 weeks old upon arrival and were group housed for ∼1 week after arrival to acclimate prior to surgery. After surgery, all rats were single housed. During recovery, rats had *ad libitum* access to standard laboratory chow and water in their home cages and were maintained on a reverse 12 h light/dark cycle. One week prior to starting behavioral training, rats began food restriction and were maintained at 85% of their free-feeding weight, although water was freely accessible in the home cage throughout the experiment. Weights were recorded throughout food restriction. All procedures were approved by the OHSU Institutional Animal Care and Use Committee and followed NIH guidelines outlined in the Guide for the Care and Use of Laboratory Animals. From a total of eight male and seven female rats assigned to experimental groups, four were excluded after histology due to misplaced intracranial cannulas.

### Surgery

#### Intracranial virus infusion and fiber-optic cannula implantation

**Figure 1.**
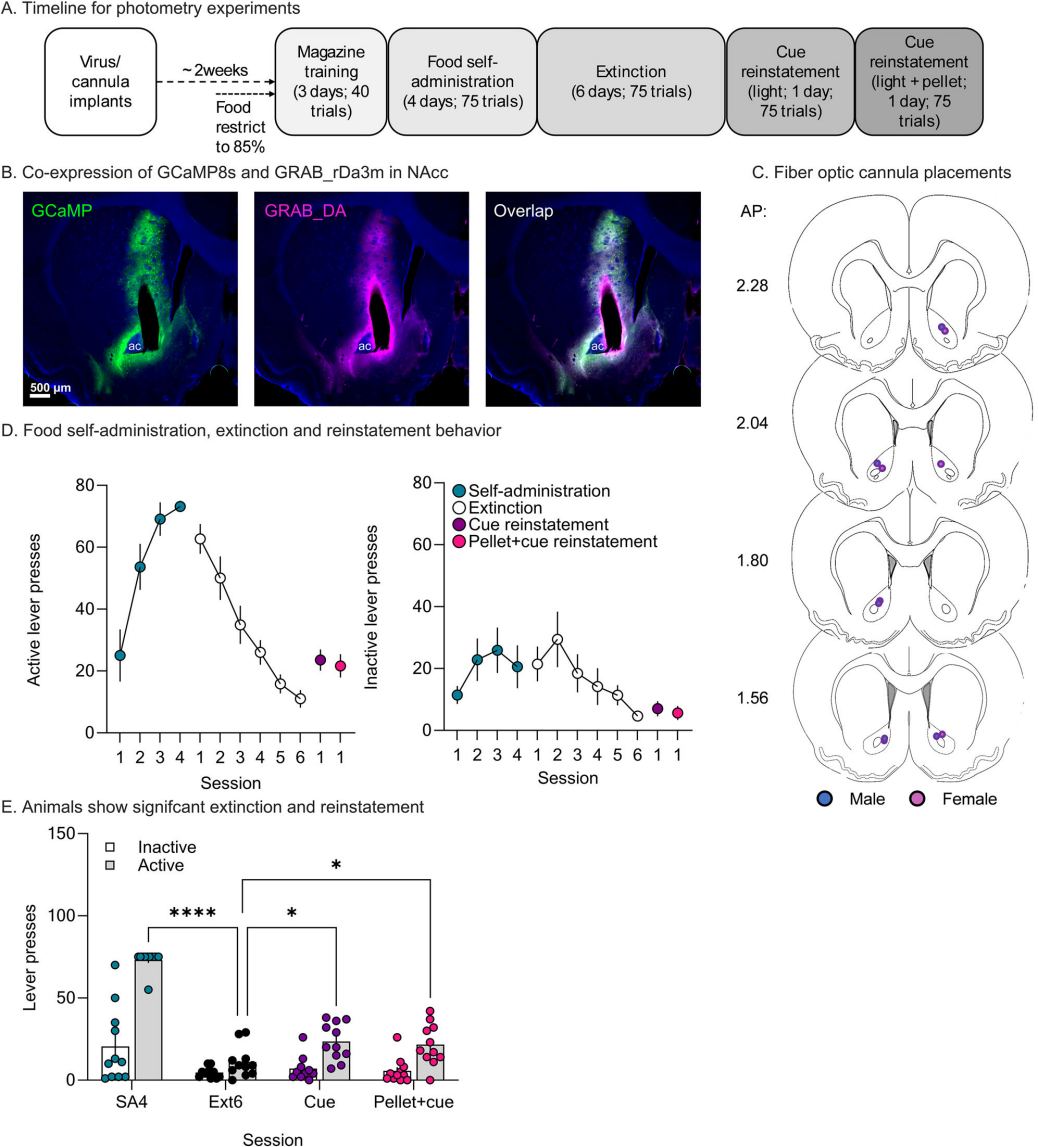
Behavior and virus expression during food self-administration training, extinction training, and reinstatement testing. ***A***, Timeline for experiment. Behavioral and fiber photometry data are reported for adult Long–Evans rats (6 male, 5 female). ***B***, Representative image of virus coexpression: left, AAV9-syn-jGCaMP8s-WPRE; middle, AAV2/9-hsyn-rDA3m; and right, overlap of viral expression. The cannula track appears as an oblong shape terminating to the right of the anterior commissure. ***C***, Fiber-optic cannula placement for rats. The dot signifies the end of the cannula (females, pink; males, blue). Placement was determined after immunohistochemistry for GFP and mApple to confirm virus expression at the end of the cannula. Sections adapted from Paxinos & Watson 7th edition. ***D***, Food self-administration sessions (SA1–4), extinction sessions (Ext1–6), and reinstatement tests. Left, Active lever presses for all rats (*n* = 11) across all sessions for all behavioral phases. Right, Inactive lever presses for the same sessions. ***E***, Data from ***D*** graphed to show direct comparison of SA4, Ext6, Cue-primed reinstatement, and Pellet + Cue-primed reinstatement (***p < 0.0001, *p < 0.05). Error bars indicate mean (±SEM) for each session while dots represent individual rats. AP, anterior posterior; ac, anterior commissure; NAcc, nucleus accumbens core. Full statistical output for experiments shown in this figure is presented in Table 1. Additional analyses related to data in this figure are presented in Extended Data Figure 1-1.

We performed bilateral (*n* = 8, 4 male/4 female; for these rats, one hemisphere was selected for recording) or unilateral (*n* = 3, 2 male/1 female) intracranial infusions of viral cocktail of GCaMP8s, a genetically encoded calcium indicator (AAV9-syn-jGCaMP8s-WPRE, Addgene #162374), and the red-shifted DA biosensor rGRAB_DA3m (AAV2/9-hsyn-rDA3m, Biohippo #BHV12400545), into the NAcc followed by implantation of fiber-optic cannula (Thorlabs, CFM15L10) above each infusion site. We performed unilateral surgeries on a subset of rats due to lack of supplies after shipping was delayed due to an ice storm. Briefly, rats were mounted in a stereotaxic device, and, after making an incision, the nose bar was adjusted such that the change in dorsal-ventral coordinates from lambda to bregma was <0.1 mm. We performed two infusions per hemisphere (250 nl each at 100 nl/min of working stock consisting of 3 µl GCaMP8s, 3 µl rGRAB_DA3m, and 3 µl sterile saline) using the following stereotaxic coordinates (relative to bregma): AP +1.3; ML ±2.4 mm; DV −7.2 mm for first infusion and DV −7.0 mm for the second infusion, 6° angle (Paxinos and Watson, 2014). Following the virus infusion, we implanted fiber-optic cannula (AP +1.3 mm; ML ±2.4 mm; DV −6.8 mm; 6° angle). We anchored the cannula to the skull with 1/8″ pan head sheet metal screws (Fastenere #842176107226) and dental cement (Stoelting #51458) and covered the fiber-optic cannula with dust caps (Thorlabs, CAPF) for protection. Rats received 5 mg/kg subcutaneous meloxicam (Covetrus, 6451602845, SKU #49755) as a postoperative analgesic. Rats then recovered for 7 d prior to beginning food restriction (see Fig. 1*A* for timeline).

### Operant behavior

#### Operant chambers

We trained and tested rats in Med Associates operant chambers (ENV-008-VPX) enclosed in sound-attenuating chambers. Each operant chamber was equipped with a nonretractable lever that served as the inactive lever (#ENV-110M) to the left of the magazine (ENV-200R2M-6.0). To the right of the magazine was a retractable lever (ENV-112CM) with a LED stimulus light (ENV-221M) above it that served as the active lever. The magazine contained a photobeam array (ENV-254-CB) to detect head entries and was connected via polyethylene tubing (Everbilt, HKP001-PVC012) to a pellet dispenser (ENV-203M-45) outside the operant chamber.

#### Magazine training

We trained rats to retrieve food from a magazine in the operant chamber for 3 d for 40 trials per day (∼20 min/day). The start of training was indicated by the onset of white noise. After a variable amount of time (ITI average = 35 s), two highly palatable food pellets (LabDiet 5TUL) were delivered into the magazine paired with a 4 s light cue. On the last day of magazine training, we habituated the rats to the fiber-optic cable. They were connected to a fiber-optic patch cable (Thorlabs Custom: FP-400URT, 0.5NA, 0.4 m length, FT023SS tubing with a 2.5 mm stainless steel ferrule) via a ceramic connector (Thorlabs, ADAF1-5), and the cable was passed through the top of the box and attached to a steel arm with a counterweight (Med Associates, PHM-110-SAI). We recorded during this last session to verify a photometry signal (see below) and assess signals related to consumption of a “free” pellet (see Results, Photometry analysis of SA sessions).

#### Food self-administration (SA)

We trained rats to self-administer food pellets during four daily sessions (SA1–SA4, ∼55 min/day for 4 d) with each session containing 75 trials. Like magazine training, the start of the session was indicated by the onset of white noise. Following this, the start of a trial was indicated by the extension of the active lever into the operant chamber. The lever was kept extended for 20 s or until the rat pressed. If the rat responded on the active lever, it immediately retracted and resulted in the delivery of two pellets and start of a 4 s light cue above the lever. If a rat failed to respond within 20 s, the lever was retracted and there was no consequence. The intertrial time was variable with an average of 25 s (minimum 20 s, maximum 30 s). Both active lever presses and magazine entries were recorded as our measure of food SA acquisition. Responses on the inactive lever were recorded but had no consequence. During magazine and SA training, water was provided *ad libitum* in the operant chamber. Photometry measures were made throughout all sessions as detailed below (Fiber photometry recordings).

#### Extinction training

Extinction training (Ext) began the day after SA4 and was identical to food SA, except that active lever presses no longer resulted in pellet delivery or cue light presentation. Six daily sessions (75 trials/session) were performed (Ext1–6).

#### Reinstatement tests

All rats received two reinstatement tests after completion of extinction training. The first test (cue-primed reinstatement; performed on the day after Ext6) began with the onset of white noise and a 4 s cue light presentation. Subsequently, the trial began with lever extension; during the trial, active lever presses resulted in cue light illumination but no pellet delivery. On the next day, rats underwent a second reinstatement test that was identical to the first except that the initial priming event at the start of the session entailed delivery of a pellet plus presentation of the light cue (pellet + cue reinstatement). We included this to see if reward + cue prime would produce a more robust effect than priming with cue alone.

#### Behavioral analysis

Both active and inactive lever presses on the last day of SA (SA4), last day of extinction (Ext6), cue reinstatement, and pellet + cue reinstatement were compared via mixed effects analysis performed in GraphPad Prism (version 10.2.2) with an assumption of sphericity (equal variability of differences). To test for an effect of session on lever pressing, we set fixed effects of session (SA4, Ext6, Cue, Pellet + cue) and lever (active or inactive), with a random effect of subject. We made simple effect comparisons comparing active and inactive responding across sessions separately and corrected for multiple comparisons using a Holm–Šídák correction. Familywise alpha threshold for confidence was set to 0.05. Full model output can be found in Table 1. Possible sex differences were not analyzed as this study only contained 11 total subjects and therefore was not powered to detect sex effects.

### Fiber photometry recordings

#### Recording parameters

Fiber photometry was performed mainly as described in a previous study using DA sensors alone (Weber et al., 2024). Prior to all recordings, fiber-optic cables were bleached at 200 mA for 8 h overnight. On the morning of the recording day, the LED power level and DC were adjusted so that the 488 nm LED, 405 nm LED, and 560 nm LED all had a power output of 40 µW at the end of the fiber-optic patch cable as measured using a Thorlabs power meter (PM100D+). For recordings, we passed excitation wavelengths (488, 405, and 560 nm) from the TDT RZ10x system via TDT patch cables (200 µm core, 2 m length, 0.5 NA). These TDT cables then connected to a Doric Minicube (FMC6) which in turn was coupled to two Thor fiber-optic patch cables*.* Emissions (555–570,460–490, and 580–680 nm) were received via the same patch cable and then decoupled at the Minicube and returned to the RZ10x via TDT response cables (600 µm, 2 m length, 0.5 NA). We acquired these emissions via the RZ10x photodetectors, digitized at 6 kHz (sampling rate), and recorded in the TDT Synapse Software (version 95-44132P) on WS4 at frequency 330 Hz for 465 nm, 210 Hz for 405 nm, and 450 Hz for 560 nm (lock-in demodulation). All recordings had a 6 Hz low-pass filter and a 9.5 V clip threshold. During behavior sessions, photometry recording was continuous for the duration of the session (all 75 trials; variable ITI of ∼52–55 min).

#### Data analysis

Fiber photometry data were analyzed using the analysis suite GuPPy created by the Lerner Lab (Northwestern University), which can be downloaded on their GitHub page. The details of the data manipulations used to generate the GCaMP8s and rGRAB_DA3m signal traces were previously described (Sherathiya et al., 2021). In brief, for each recording session, we applied least-squares linear fit to align the same 405 nm isosbestic channel to both DA and calcium signal channels and used fluctuations in the isosbestic channel to account for florescence changes in the signal channels that are not a result of ligand binding. Any disconnects during the recording, defined by a rapid substantial shift (>10 mV) in average mV, were snipped prior to fitting the traces and any lever presses within those snips were excluded from further analysis. Then we calculated a change in fluorescence measure (Δ*F*/*F* = Signal − Fitted Control / Fitted Control) and applied *z*-score normalization to control for between-session and between-rat differences in virus expression or recording. Finally, we averaged normalized traces across behavioral event replicates for each rat and then averaged by session. For raw demodulated data, we extracted data traces with the TDT python package and graphed using Matplotlib with code adapted from python code from previous publications (Lerner et al., 2015; Barker et al., 2017).

For DA and calcium transients associated with behavioral events of interest, first we extracted *z*-scored Δ*F*/*F* traces within a 15 s window around each behavioral event and averaged all traces to generate an average response for the rat during each test, using −5 to 0 s (relative to the event) as a baseline period for normalization. We identified significant DA and calcium transients using continuous threshold bootstrapping methods (Jean-Richard-Dit-Bressel et al., 2020). We selected a consecutive threshold of 1,000 for our 6 kHz acquisition, which equates to 0.167 s, based on recommendation from the code developer given our sampling rate. Using this analysis, we identified instances in our recorded traces where the *z*-score is 95% likely to not be at baseline and marked these epochs with horizontal bars above the traces. In addition to this analysis, we performed permutation tests on the bootstrapped distributions.

In tandem with this analysis, we also performed the classical hypothesis-driven approach in which we selected time windows before and after our behavioral event of interest and calculated the area under the curve (AUC) for the *z*-scored ΔF/F trace within this time window. Using these AUC values, we performed two-tailed paired *t* tests comparing SA1 versus SA4, Ext01 versus Ext06, and Ext06 versus each reinstatement test. We assumed a Gaussian distribution for all *t* tests and set our confidence level to 95%.

For all correlations of photometry data and behavior, we used a simple linear regression in GraphPad Prism 10.6.0.

#### Methodological considerations for sensor multiplexing

Despite simultaneously recording both MSN calcium and DA, we did not make direct quantitative comparisons due to considerations detailed here. First, while we selected GCaMP8s and rGRAB_DA3m due to their reported brightness and similar kinetics, there are small differences in kinetics which could impact our results (Zhang et al., 2023; Zhuo et al., 2023). Furthermore, measurements of these parameters are made typically in cultured neurons with controlled amounts of ligand. All our recordings are made in vivo and of particular note, measure changes taking place in either an intracellular (calcium) or extracellular (DA) environment. The relative abundance of calcium or DA can change the kinetics of these sensors, so we avoided comparing onset and offset of calcium and DA directly. Another consideration is that we normalized to baseline in all our peri-event analyses and the level of calcium or DA at our defined baseline could affect subsequent analyses. For instance, if extracellular DA is very low during baseline then any phasic release will show as a prominent peak, whereas if there is more baseline activity for calcium, then any phasic increase or decrease may be harder to detect. This makes direct comparisons of the two types of transients difficult to interpret, leading us to limit our interpretations of the relationship between DA and calcium signals.

Another challenge of sensor multiplexing, particularly with green and red channels, is that many red fluorophores show emission when excited with blue light. We used a blue light channel to record an isosbestic signal for our green channel, but this light was consistently applied throughout recording and therefore no transient changes should be due to blue light excitation. However, a consideration with the isosbestic channel is signal bleed from the green GCaMP into the red GRAB_DA. We applied the same blue isosbestic recording (405 nm excitation wavelength) to both green and red channels, and it is known that, with a strong green signal, negative peaks may appear in the isosbestic recording as 405 nm is not the true isosbestic point of most GFP biosensors. Thus, when creating the Δ*F*/*F* signal, it is possible that peaks or dips are slightly amplified. When comparing isosbestic and signal channels for the same sensor, this is typically not an issue but given that we applied a blue isosbestic recording to a different sensor, it is possible some of the GCaMP signal may be reflected in our GRAB_DA traces. We believe this to be unlikely given how different these transients are in shape and in magnitude across different phases of the experiment. However, we did examine the raw fluorescence data for all recorded channels, and there appears to be little influence of the 405 signal on the 560 signal (see representative trace of a full SA4 session as well as time-locked average for all rats in Extended Data Fig. 3-1). However, the uncorrected data are difficult to compare; therefore in addition to visualizing the raw data, we processed a subset of data without the inclusion of an isosbestic channel, instead of using a fitted exponential curve for the ΔF/F subtraction, and the data appeared largely unchanged (Extended Data Fig. 3-2; Extended Data Tables 3-1, 3-2).

### Histology

Upon completion of experiments, rats were killed via lethal injection of Fatal Plus (Covetrus, #35946) diluted to 80 mg/kg with sterile saline. Once animals no longer displayed reflexive motor responses, they were perfused transcardially first with 1× phosphate-buffered saline (PBS) followed by 4% formaldehyde/1% methanol in 1× PBS, pH ∼7, at a rate of ∼100 ml over 5 min. Brains were extracted and allowed to rest in the formaldehyde solution for up to 24 h. Brains were transferred to 1× PBS with 0.01% sodium azide and then sliced on a vibratome (Leica VT1000s; frequency 8, speed 70, blade DORCO plat. 5T300) at 60 µm. Slices were kept in a 24-well plate in 1× PBS with 0.01% sodium azide at 4°C until the start of immunohistochemistry (IHC).

#### Immunohistochemistry

We performed IHC on tissue from photometry experiments to amplify the GFP and mApple signals prior to imaging to confirm virus expression and placement. We began IHC with three 30 min washes, first in 1× PBS (diluted from 10× PBS, Quality Biological, 119-069-151) and then twice in 1× PBS with 0.5% (v/v) Triton X-100 (Electron Microscopy Sciences, #22140). After this we permeabilized the tissue for 2 h in 1× PBS with 0.5% (v/v) Triton X-100, 20% (v/v) DMSO (Sigma-Aldrich, #276855), and 2% (w/v) glycine (Sigma-Aldrich, #G8898) at room temperature (RT) on a rocking shaker. Next, we blocked tissue in 1× PBS with 0.5% (v/v) Triton X-100, 10% (v/v) DMSO, and 6% (v/v) Normal Donkey Serum (NDS, Jackson ImmunoResearch, 017-200-121) for 2 h at RT on a rocking shaker. After blocking we incubated the tissue overnight at RT in 1:1,000 Anti-GFP (Aves, GFP-1010) and 1:1,000 Anti-DsRed2 (Santa Cruz Biotechnology, SC-101526) in 1× PBS with 0.5% (v/v) Tween 20, and 0.01% (w/v) Heparin (Sigma-Aldrich, #H3393-100KU) with 3% NDS and 10% DMSO on a rotator. The following day, we washed the tissue three times for 30 min in 1× PBS with 0.5% (v/v) Tween 20 (Thermo Scientific, #J20605-AP) and 0.01% (w/v) heparin before incubating in 1:250 Anti-chicken-488 (Jackson ImmunoResearch, 775-546-155) and Anti-mouse-647 (Jackson ImmunoResearch, 715-606-150) in 1× PBS with 0.5% (v/v) Tween 20 and 0.01% (w/v) heparin with 3% NDS overnight at RT on a rotator. On the third day, we performed two 30 min washes in 1× PBS with 0.5% (v/v) Tween 20 and 0.01% (w/v) heparin before beginning a final wash in 1× PBS (30 min). Slices were kept in 1× PBS with 0.01% sodium azide at 4°C until being mounted onto Superfrost Plus slides (Fisher Scientific, catalog #1255015) and coverslipped (Fisher Scientific, 12541026) using Vectashield Vibrance with DAPI (Vector Labs, H-1800-10).

#### Imaging

Images were acquired using a Leica DMi8 inverted microscope equipped with an ORCA-Flash4.0 LT+ Digital CMOS camera (Hamamatsu). LASX Premium Software was used for image acquisition and ImageJ was used for analysis.

#### Experimental design and statistical analyses

**Figure 2.**
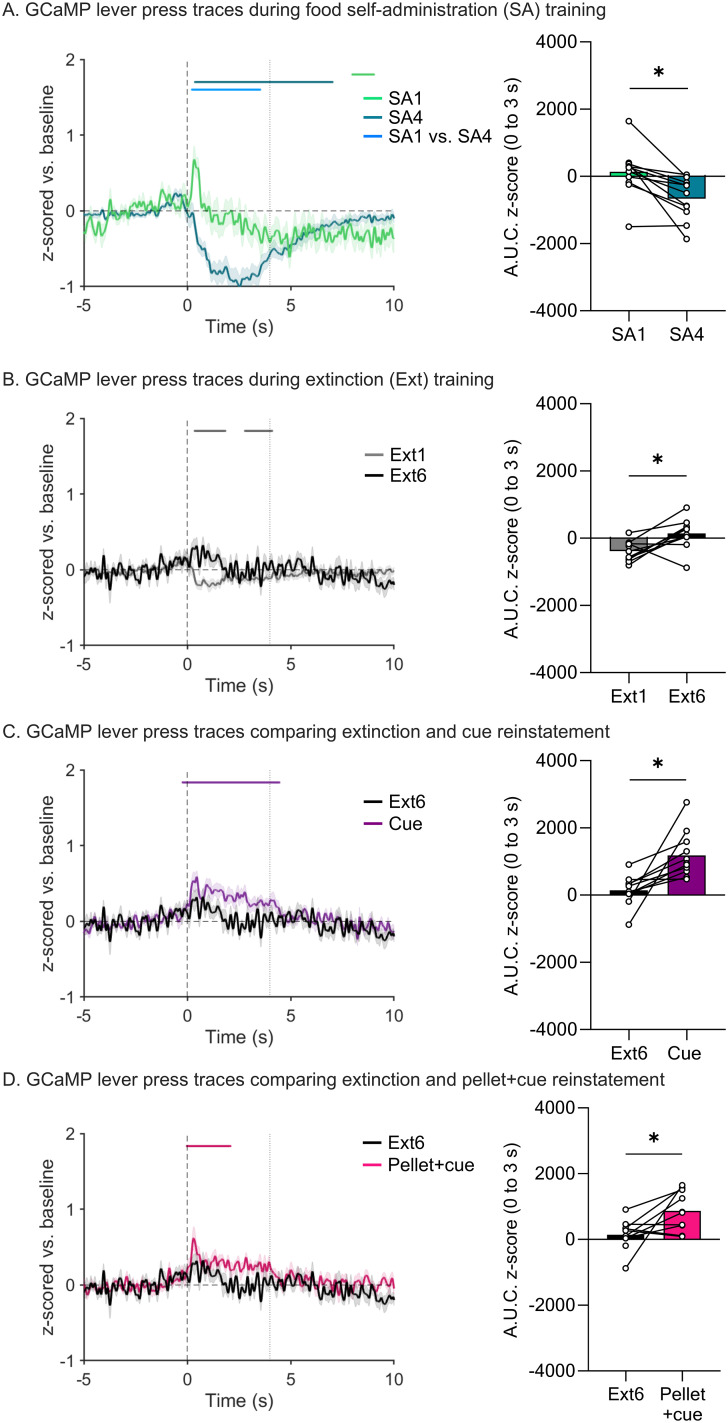
Fiber photometry recordings of NAcc GCaMP signals across food self-administration, extinction, and reinstatement sessions. ***A***, Left, *z*-scored mean GCaMP traces time-locked to active lever presses in first session of self-administration (SA1) and the last session of self-administration (SA4) normalized to a baseline period (−5 to 0 s). SEM is shown in shaded area around the mean. The black vertical dashed line at time 0 s indicates the lever press with the gray vertical dashed line at time 4 s indicating the end of the light cue. The matching-colored lines above the traces show periods in the 15 s window during which bootstrapping indicates 95% confidence that the mean is not equal to zero (baseline level). The light blue lines above the traces indicate time periods in which the bootstrapped confidence intervals are significantly different from each other. Right, Area under the curve for the traces shown in the left. Bars show mean (±SEM) for 3 s after the lever press while dots indicate individual rats with lines connecting individual between-session comparisons. ***B–D***, These panels show GCaMP traces, as described in ***A***, during the first session of extinction (Ext1) and the last session of extinction (Ext6; ***B***), the last session of extinction (Ext6) and cue-primed reinstatement (Cue; ***C***), and the last session of extinction (Ext6) and pellet + cue-primed reinstatement (Pellet + cue; ***D***). Full statistical output for experiments shown in this figure is presented in Tables 2 and 3. Additional analyses related to data in this figure are presented in Extended Data Figures 2-1–2-4, with statistical output for these extended data figures presented in Extended Data Tables 2-1–2-4.

**Figure 3.**
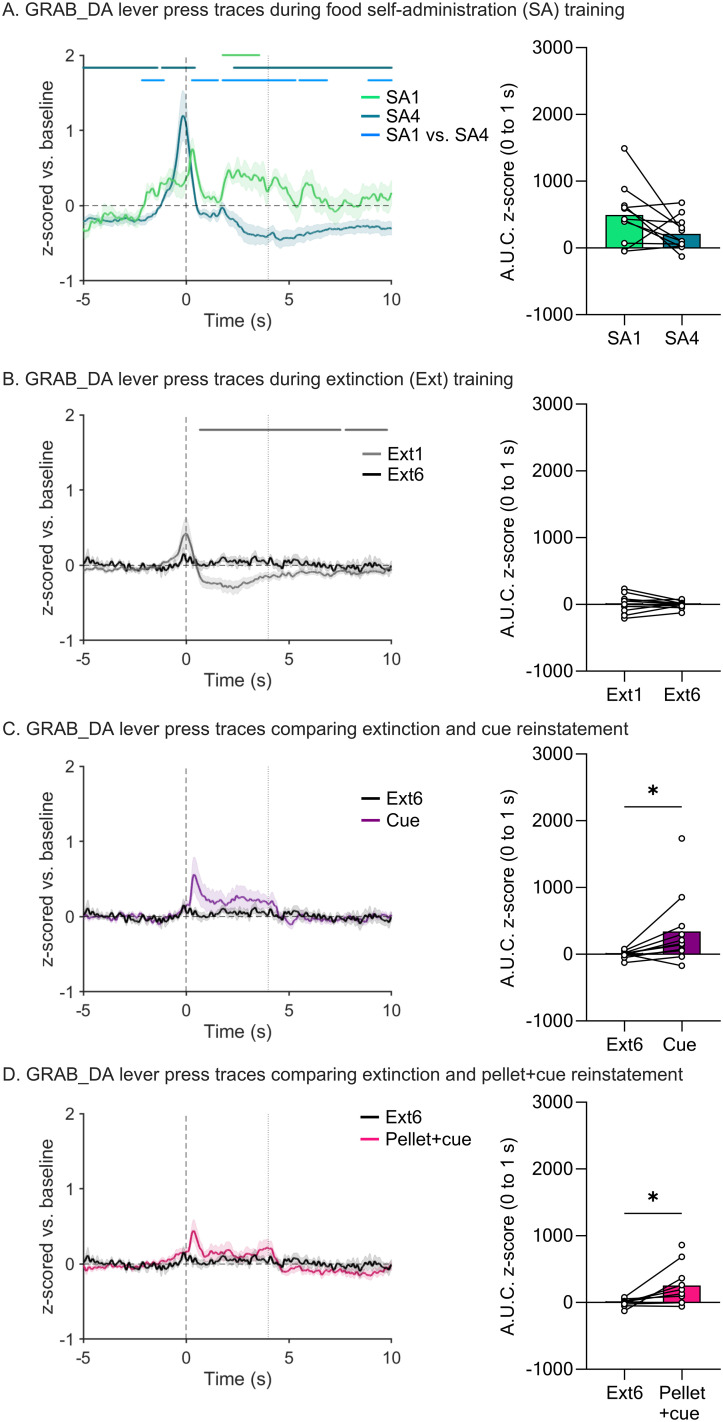
Fiber photometry recordings of GRAB_DA signals in the NAcc across self-administration, extinction, and reinstatement sessions. ***A***, Left, *z*-scored mean DA traces time-locked to active lever presses in first session (SA1) and last session (SA4) of self-administration normalized to a baseline period (−5 to 0 s). SEM is shown in shaded area around the mean. Black, vertical dashed line at time 0 s indicates the lever press. Gray, vertical dashed line at time 4 s indicates the end of the light cue. The matching-colored lines above the traces identify significant transients, i.e., periods in the 15 s window (−5 to 10 s) during which bootstrapping indicates 95% confidence that the mean is not equal to zero (baseline level). The light blue lines above the traces indicate time periods in which the bootstrapped confidence intervals are significantly different from each other. Right, Area under the curve for the traces shown in the left. Bars show mean (±SEM) AUC for the first 1 s after the lever press while dots indicate individual rats with lines connecting individual between-session comparisons. ***B–D***, These panels show GRAB_DA traces, as described in ***A***, during the first session of extinction (Ext1) and the last session of extinction (Ext6; ***B***), the last session of extinction (Ext6) and cue-primed reinstatement (Cue; ***C***), and the last session of extinction (Ext6) and pellet + cue-primed reinstatement (Pellet + cue; ***D***). Area under the curve for these traces is shown on the right. *p < 0.05. Full statistical output for experiments shown in this figure is presented in Tables 4 and 5. Additional analyses related to data in this figure are presented in Extended Data Figures 3-1–3-4, with statistical output for these extended data figures presented in Extended Data Tables 3-1–3-5.

**Figure 4.**
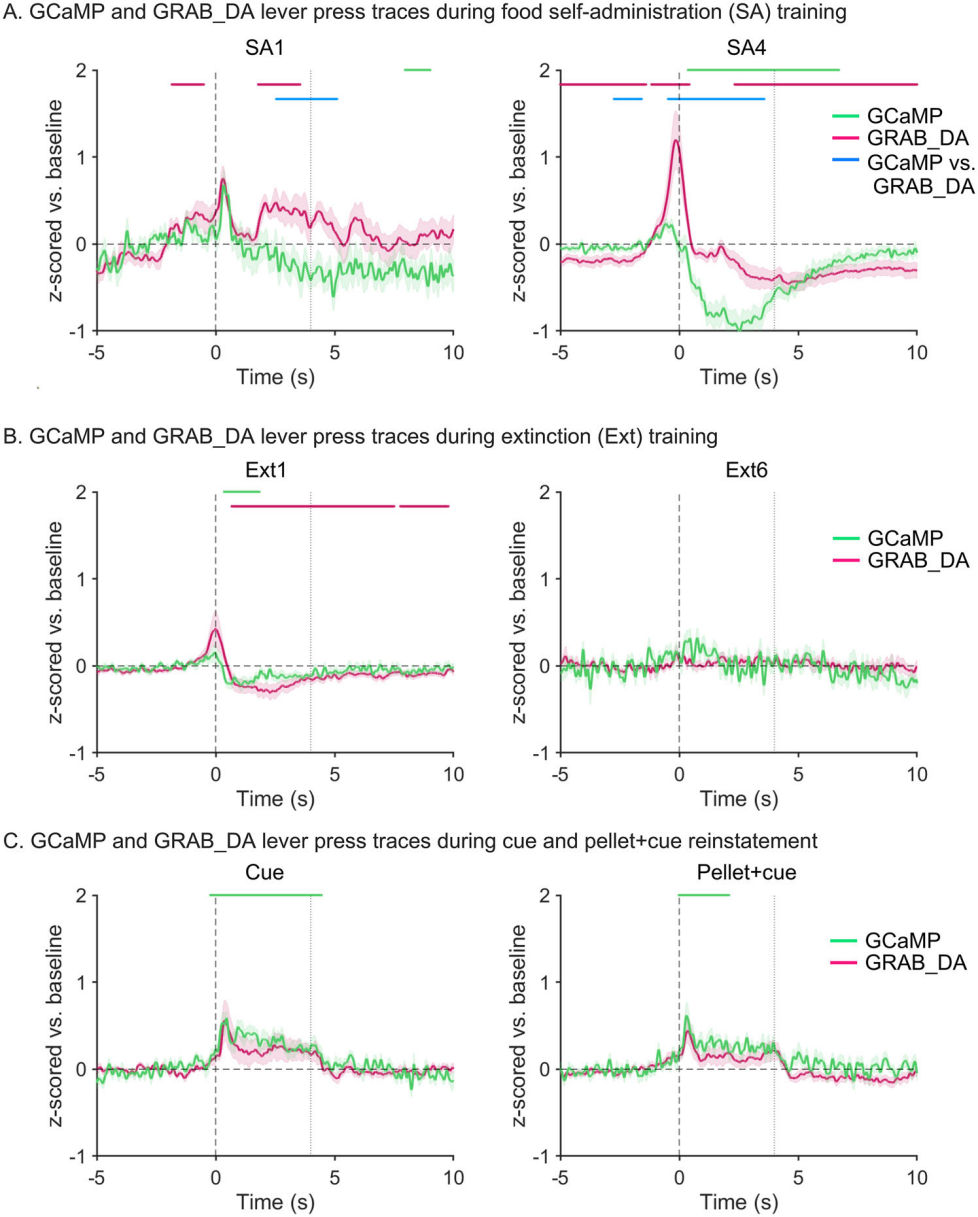
Fiber photometry recordings of GRAB_DA and GCaMP transients in the NAcc across self-administration, extinction, and reinstatement sessions. ***A***, Left, *z*-scored mean traces time-locked to active lever presses in first self-administration session (SA1) normalized to a baseline period (−5 to 0 s). SEM is shown in shaded area around the mean. Black vertical dashed line at time 0 s indicates the lever press. Gray vertical dashed line at time 4 s indicates the end of the light cue. The matching-colored lines above the traces identify significant transients, i.e., periods in the 15 s window (−5 to 10 s) during which bootstrapping indicates 95% confidence that the mean is not equal to zero (baseline level). The light blue lines above the traces indicate time periods in which the bootstrapped confidence intervals are significantly different from each other. Right, *z*-scored mean traces for both DA and calcium during SA4 as described above for SA1. ***B***, ***C***, These panels show traces, as described in ***A***, during the first session of extinction (Ext1) and the last session of extinction (Ext6; ***B***), and cue-primed reinstatement (Cue) and pellet + cue-primed reinstatement (Pellet + cue; ***C***). Full statistical output for experiments shown in this figure is presented in Table 6.

Adult Long–Evans rats (6 male, 5 female) received stereotaxic infusions into NAcc of viruses expressing calcium and DA sensors followed by implantation of a fiber-optic cannula above the site of virus infusion. Fiber photometry recordings were performed on each day of self-administration training (rats lever-pressed to receive a palatable food pellet paired with a cue), each day of extinction training, and during two tests for reinstatement. Brains were collected to verify virus expression and localization. Details of all procedures are provided above, and the experimental timeline is depicted in Figure 1*A*. Statistical output for each main text and supplemental figure is provided in the tables that list statistical tests employed, comparisons made, and *t* and/or *p* values (significance set at *p* < 0.05). For Figure 1, statistical output for behavioral data is presented in Table 1. For Figure 2, statistical output for photometry data is presented in Tables 2 and 3. For Figure 3, statistical output for photometry data is presented in Tables 4 and 5. For Figure 4, statistical output of photometry data is presented in Table 6. For extended data figures, all statistical output can be found in the extended data tables. Additional information on statistical analysis is presented above in sections titled Operant behavior, Behavioral analysis and Fiber photometry recordings, Data analysis.

## Results

Two weeks prior to the start of behavioral testing, rats received either bilateral (*n* = 8) or unilateral infusions (*n* = 3) of a viral cocktail of GCaMP8s and GRAB_rDA3m into the NAcc. In the same surgery, a fiber-optic cannula was implanted above the virus injection site. Rats were allowed to recover for 1 week and then experienced 1 week of restricted chow access in the home cage (Fig. 1*A*). A representative image of virus coexpression of GCaMP8s and GRAB_DAr3m (showing the cannula track) is provided in Figure 1*B*, whereas cannula placements are summarized in Figure 1*C*.

10.1523/ENEURO.0380-25.2026.f1-1 Figure 1-1 Area under the curve (AUC) for GCaMP and GRAB_DA traces across all recorded sessions (n = 5 female and 6 male Long-Evans rats). **A**. Behavioral data shown in Fig. 1 are duplicated here for comparison to AUC values. **B.** GCaMP AUC data (0-3 s) for all rats across all behavioral sessions. Bars show mean (±SEM) AUC while dots indicate individual rats. **C**. GRAB_DA AUC data (0-1 s) across all behavioral sessions, presented as described for panel **B**. Download Figure 1-1, TIF file.

### Behavioral results

We trained rats to lever press for delivery of two highly palatable pellets paired with a 4 s light cue. Rats readily acquired this behavior, with the majority reaching 100% trial completion (75 trials) after 4 d of SA training (Fig. 1*D*; this panel shows responding during SA training and also during extinction training and reinstatement tests). We then put rats through extinction training in which active lever presses no longer resulted in a food reward or a light cue. After six sessions of extinction, rats had suppressed their responding on the active lever compared with the last day of self-administration (Fig. 1*E*; *t*_(59)_ = 14.77, *p* < 0.0001). They also reduced responding on the inactive lever, a permanently extended lever associated with no consequence throughout all training and testing (Fig. 1*E*; *t*_(59)_ = 3.779, *p* = 0.0022). Once lever pressing had been extinguished, we tested the rats for cue-primed reinstatement. The session began with a single noncontingent presentation of the light, and during the remainder of this ∼55 min test, lever presses delivered the same 4 s light cue previously paired with food reward. Rats increased responding for the cue compared with the last day of extinction (Fig. 1*E*; *t*_(59)_ = 2.980, *p* = 0.0125) with no effect on inactive lever responding. On the next day, we performed a second reinstatement test identical to the first except that the initial light cue also was paired with noncontingent delivery of a pellet, acting as a reward + cue prime. As observed in the previous reinstatement test, rats exhibited increased responding on active but not inactive levers (Fig. 1*E*; *t*_(59)_ = 2.572, *p* = 0.0282). See Table 1 for the full statistical output.

**Table 1.** Statistical output for behavioral data in Figure 1

Expt phase	Measure	Fixed effects	*F* value	*p* value	Significant?	Figure
SA4, Ext6, Cue, Pellet + cue	Lever presses (*n* = 11, 6M/5F)	Session × Lever				1*E*
		Session	*F*_(3,30)_ = 68.67	<0.0001	****	
		Lever	*F*_(1,10)_ = 88.86	<0.0001	****	
		Session × Lever	*F*_(3,29)_ = 23.85	<0.0001	****	
		Holm-Šídák^a^				
		Inactive				
		SA4 vs Ext6	*t*_(59)_ = 3.779	0.0022	**	
		SA4 vs Cue	*t*_(59)_ = 2.995	0.0159	*	
		SA4 vs Pellet + cue	*t*_(59)_ = 3.542	0.0039	**	
		Ext6 vs Cue	*t*_(59)_ = 0.6811	0.8739	n.s.	
		Ext6 vs Pellet + cue	*t*_(59)_ = 0.2376	0.8805	n.s.	
		Cue vs Pellet + cue	*t*_(_*_5_*_9)_ = 0.4500	0.8805	n.s.	
		Active				
		SA4 vs Ext6	*t*_(59)_ = 14.77	<0.0001	****	
		SA4 vs Cue	*t*_(59)_ = 11.79	<0.0001	****	
		SA4 vs Pellet + cue	*t*_(59)_ = 12.24	<0.0001	****	
		Ext6 vs Cue	*t*_(59)_ = 2.980	0.0125	*	
		Ext6 vs Pellet + cue	*t*_(59)_ = 2.527	0.0282	*	
		Cue vs Pellet + cue	*t*_(59)_ = 0.4535	0.6518	n.s.	

a Adjusted *p* values reported for post hoc comparisons. M, male, F, female.

### Photometry overview

We simultaneously recorded both MSN calcium transients (Fig. 2) and DA release (Fig. 3) in the NAcc on all days of the regimen. For all traces, we examined a 15 s window around the active lever press. GCaMP and DA traces are plotted to compare the first and last day of food SA (Figs. 2*A*, 3*A*), the first and last day of extinction (Figs. 2*B*, 3*B*), the last day of extinction and the cue-primed reinstatement (Figs. 2*C*, 3*C*), and the last day of extinction and the pellet + cue-primed reinstatement (Figs. 2*D*, 3*D*). Above all the traces are horizontal lines of matching color that indicate periods where the 95% confidence interval does not cross baseline (i.e., *z*-score = 0) for the consecutive threshold period determined with a continuous threshold bootstrapping method (Jean-Richard-Dit-Bressel et al., 2020; Liu et al., 2020; Yau and McNally, 2022). Time periods during which permutation testing revealed differences between sessions are indicated by a light blue line. Sections that follow will separately address data obtained for SA, extinction, and reinstatement phases of the experiment, but a summary of GCaMP and GRAB_DA data across all phases is provided in Extended Data Figure 1-1.

10.1523/ENEURO.0380-25.2026.f2-1 Figure 2-1 Fiber photometry recordings of GCaMP transients in the NAcc associated with “free” pellet consumption during magazine training. GCaMP z-scored mean traces time-locked to magazine entries in the third magazine training session (MagTrain) and the fourth self-administration session (SA4) normalized to a baseline period (-5 to 0 s). SEM is shown in shaded area around the mean. Black vertical dashed line at time 0 s indicates the magazine entry. The matching-colored line above the traces identifies a significant transient for SA4, i.e., a period in the 15-s window (-5 to 10 s) during which bootstrapping indicates 95% confidence that the mean is not equal to zero (baseline level). Full statistical output for experiments shown in this figure is presented in Table 2-1. Download Figure 2-1, TIF file.

10.1523/ENEURO.0380-25.2026.f2-2 Figure 2-2 Fiber photometry recordings of NAcc GCaMP signals across food self-administration, extinction, and reinstatement sessions focusing on first lever press, first five lever presses, or the first 10 min of key sessions. **A.** z-scored mean GCaMP traces time-locked to the first active lever press in the first session of self-administration (SA1) and the last session of self-administration (SA4) normalized to a baseline period (-5 to 0 s). SEM is shown in shaded area around the mean. The black vertical dashed line at time 0 s indicates the lever press while the gray vertical dashed line at time 4 s indicates the end of the light cue. The matching-colored lines above the traces show periods in the 15-s window during which bootstrapping indicates 95% confidence that the mean is not equal to zero (baseline level). The light blue lines above the traces indicate time periods in which the bootstrapped confidence intervals are significantly different from each other. **B.** z-scored mean GCaMP traces time-locked to the first five active lever presses of SA1 and SA4. **C**. z-scored mean GCaMP traces time-locked to the active lever presses in first 10 min of SA1 and SA4. **D-L.** These panels show GCaMP traces, as described in **A-C,** during the first session of extinction (Ext1) and the last session of extinction (Ext6) (**D-F**), the last session of extinction (Ext6) and cue-primed reinstatement (Cue) (**G-I**), and the last session of extinction (Ext6) and pellet+cue-primed reinstatement (Pellet+cue) (**J-L)**. Full statistical output for experiments shown in this figure is presented in Table 2-2. Download Figure 2-2, TIF file.

10.1523/ENEURO.0380-25.2026.f2-3 Figure 2-3 Fiber photometry recordings of GCaMP signals in the NAcc time-locked to lever entry across self-administration, extinction, and reinstatement sessions. **A.** *Left:* z-scored mean GCaMP traces time-locked to lever entry in first session (SA1) and last session (SA4) of self-administration normalized to a baseline period (-5 to 0 s). SEM is shown in shaded area around the mean. Black, vertical dashed line at time 0 s indicates the lever entry. The matching-colored lines above the traces identify significant transients, i.e., periods in the 15-s window (-5 to 10 s) during which bootstrapping indicates 95% confidence that the mean is not equal to zero (baseline level). The light blue lines above the traces indicate time periods in which the bootstrapped confidence intervals are significantly different from each other. *Right:* Area under the curve for the traces shown in *Left*. Bars show mean (±SEM) for 3 s after the lever press while dots indicate individual rats with lines connecting individual between session comparisons. **B-D.** These panels show GCaMP traces, as described in **A,** during the first session of extinction (Ext1) and the last session of extinction (Ext6) (**B**), the last session of extinction (Ext6) and cue-primed reinstatement (Cue) (**C**), and the last session of extinction (Ext6) and pellet+cue-primed reinstatement (Pellet+cue) (**D)**. Area under the curve for these traces is shown on the *Right*. *p<0.05. Full statistical output for experiments shown in this figure is presented in Tables 2-3 and 2-4. Download Figure 2-3, TIF file.

10.1523/ENEURO.0380-25.2026.f2-4 Figure 2-4 Latency to lever press and simple linear regression analysis assessing the relationship between latency and Area Under the Curve (AUC) of GRAB_DA or GCaMP traces across behavioral sessions. **A.** Average latency to lever press from time of lever entry for all rats across behavioral sessions. Bars show mean (±SEM) while dots indicate individual rats. **B.** Simple linear regression analysis comparing AUC values for GCaMP and GRAB_DA to the average latency for SA1. AUC was determined for each lever press in the session and averaged for each rat over 0 to 3 s (GCaMP) or 0 to 1 s (GRAB_DA) (selected to correspond to time bins in Figs. 2 and 3). Full traces and AUC values are shown in Fig. 2 (GCaMP) and Fig. 3 (GRAB_DA). The linear regression line is shown in green for GCaMP and magenta for GRAB_DA on all graphs with the 95% confidence intervals indicated by dashed lines. r^2^ and p values for the regression analysis are provided within each graph and color coded to their respectively regression lines. Dots indicate individual rats. **C-G.** Same as analysis described in **B** but for SA4 (**C**), extinction (Ext) 1 (**D**), Ext6 (**E**), cue reinstatement (**F**), and pellet+cue reinstatement (**G**). Download Figure 2-4, TIF file.

10.1523/ENEURO.0380-25.2026.f3-1 Figure 3-1 Raw fluorescence data for all recorded channels. **A.** Representative traces showing raw demodulated fluorescence data for SA4 of one rat. **B.** Raw fluorescence averaged across all rats (n = 11) for SA4 for 405 nm (isosbestic), 465 nm (GCaMP), and 560 nm (GRAB_DA) channels. SEM is shown as shaded area around the mean. Black vertical dashed line at time 0 s indicates the lever press. Gray vertical dashed line at time 4 s indicates the end of the light cue. ISOS, isosbestic. Download Figure 3-1, TIF file.

10.1523/ENEURO.0380-25.2026.f3-2 Figure 3-2 Fiber photometry recordings of GRAB_DA transients in the NAcc during food self-administration (SA4) analyzed with the inclusion of the 405 nm isosbestic channel and without. **A.** *Left:* z-scored mean DA traces time-locked to active lever presses in the last session (SA4) of self-administration normalized to a baseline period (-5 to 0 s). SEM is shown as shaded area around the mean. Black vertical dashed line at time 0 s indicates the lever press. Gray vertical dashed line at time 4 s indicates the end of the light cue. The matching-colored lines above the traces identify significant transients, i.e., periods in the 15-s window (-5 to 10 s) during which bootstrapping indicates 95% confidence that the mean is not equal to zero (baseline level). *Right:* Area under the curve for the traces shown in *Left*. Bars show mean (±SEM) for time window starting at the lever press (0 s) to 1 s after, while dots indicate individual rats with lines connecting individual between session comparisons. **B.** These panels show GRAB_DA traces, as described in **A,** but for cue-primed reinstatement (Cue). Area under the curve for these traces is shown on the *Right* (0-1 s). Full statistical output for experiments shown in this figure is presented in Tables 3-1 and 3-2. ISOS, isosbestic. Download Figure 3-2, TIF file.

10.1523/ENEURO.0380-25.2026.f3-3 Figure 3-3 Fiber photometry recordings of NAcc GRAB_DA signals across food self-administration, extinction, and reinstatement sessions focusing on first lever press, first five lever presses, or the first 10 min of key sessions. **A.** z-scored mean GRAB_DA traces time-locked to the first active lever press in the first session of self-administration (SA1) and the last session of self-administration (SA4) normalized to a baseline period (-5 to 0 s). SEM is shown in shaded area around the mean. The black vertical dashed line at time 0 s indicates the lever press while the gray vertical dashed line at time 4 s indicates the end of the light cue. The matching-colored lines above the traces show periods in the 15-s window during which bootstrapping indicates 95% confidence that the mean is not equal to zero (baseline level). The light blue lines above the traces indicate time periods in which the bootstrapped confidence intervals are significantly different from each other. **B.** z-scored mean GRAB_DA traces time-locked to the first five active lever presses of SA1 and SA4. **C**. z-scored mean GRAB_DA traces time-locked to the active lever presses in first 10 min of SA1 and SA4. **D-L.** These panels show GRAB_DA traces, as described in **A-C,** during the first session of extinction (Ext1) and the last session of extinction (Ext6) (**D-F**), the last session of extinction (Ext6) and cue-primed reinstatement (Cue) (**G-I**), and the last session of extinction (Ext6) and pellet+cue-primed reinstatement (Pellet+cue) (**J-L)**. Full statistical output for experiments shown in this figure is presented in Table 3-3. Download Figure 3-3, TIF file.

10.1523/ENEURO.0380-25.2026.f3-4 Figure 3-4 Fiber photometry recordings of GRAB_DA signals in the NAcc time-locked to lever entry across self-administration, extinction, and reinstatement sessions. **A.** *Left:* z-scored mean DA traces time-locked to lever entry in first session (SA1) and last session (SA4) of self-administration normalized to a baseline period (-5 to 0 s). SEM is shown in shaded area around the mean. Black, vertical dashed line at time 0 s indicates the lever entry. The matching-colored lines above the traces identify significant transients, i.e., periods in the 15-s window (-5 to 10 s) during which bootstrapping indicates 95% confidence that the mean is not equal to zero (baseline level). The light blue lines above the traces indicate time periods in which the bootstrapped confidence intervals are significantly different from each other. *Right:* Area under the curve for the traces shown in *Left*. Bars show mean (±SEM) AUC over the first 1 s after the lever press while dots indicate individual rats with lines connecting individual between session comparisons. **B-D.** These panels show GRAB_DA traces, as described in **A,** during the first session of extinction (Ext1) and the last session of extinction (Ext6) (**B**), the last session of extinction (Ext6) and cue-primed reinstatement (Cue) (**C**), and the last session of extinction (Ext6) and pellet+cue-primed reinstatement (Pellet+cue) (**D)**. Area under the curve for these traces is shown on the *Right*. *p<0.05. Full statistical output for experiments shown in this figure is presented in Tables 3-4 and 3-5. Download Figure 3-4, TIF file.

### Photometry analysis of SA sessions

Focusing on comparison of food SA sessions 1 and 4 (SA1 and SA4), analysis of GCaMP traces with bootstrapping showed the development of a significant suppression of MSN calcium levels as training progressed (Fig. 2*A*, left; statistical analysis in Table 2). We quantified the AUC for the time period 0–3 s and compared SA1 with SA4 with a paired *t* test to confirm this decrease (Fig. 2*A*, right; *t*_(10)_ = 5.143, *p* = 0.0004; full model output in Table 3). The increased magnitude of suppression of the GCaMP signal as training progressed suggests a contribution of learning. However, there is some evidence linking reduced MSN activity to consummatory behavior (see Introduction and Discussion). As a first step toward disentangling this, we analyzed GCaMP signals associated with consumption of a “free” pellet during the last day of magazine training by time-locking to magazine entry in a subset of rats (*n* = 3). It is important to note that not all magazine entries are tied to consumption as the rats often enter the magazine even when a pellet is not present during training. However, it is interesting that a small decrease in the GCaMP signal was apparent, although bootstrapping did not identify this dip as significantly different from baseline (Extended Data Fig. 2-1, Extended Data Table 2-1). For comparison we analyzed GCaMP data from SA4 time-locked to magazine entry and superimposed this on the magazine training data (Extended Data Fig. 2-1). The much greater suppression of the GCaMP signal during SA4 compared with magazine training, combined with the increase in magnitude of suppression from SA1 to SA4, supports the idea that dips below baseline do not exclusively reflect consummatory behavior.

**Table 2.** Statistical output for bootstrapping analyses in Figure 2

Expt phase	Measure	Factors in analysis	Time 95% CI ≠ 0	Significantly different?	Figure
SA	GCaMP response to lever press, *z*-scored trace (*n* = 11)	Bootstrapping		0.235–3.51 s	2*A*, left
		SA1	8.10–9.01 s		
		SA4	0.332–6.69 s		
Extinction	GCaMP response to lever press, *z*-scored trace (*n* = 11)	Bootstrapping		n.s.	2*B*, left
		Ext1	0.422–1.83 s, 2.82–4.10 s		
		Ext6	n.s.		
Extinction/reinstatement	GCaMP response to lever press, *z*-scored trace (*n* = 11)	Bootstrapping		n.s.	2*C*, left
		Ext6	n.s.		
		Cue test	−0.222 to 4.45 s		
Extinction/reinstatement	GCaMP response to lever press, *z*-scored trace (*n* = 11)	Bootstrapping		n.s.	2*D*, left
		Ext6	n.s.		
		Pellet + cue test	0–2.05 s		

10.1523/ENEURO.0380-25.2026.t2-1 Table 2-1 Statistical output for bootstrapping analyses in Figure 2-1 Download Table 2-1, DOCX file.

10.1523/ENEURO.0380-25.2026.t2-2 Table 2-2 Statistical output for bootstrapping analyses in Figure 2-2 Download Table 2-2, DOCX file.

10.1523/ENEURO.0380-25.2026.t2-3 Table 2-3 Statistical output for bootstrapping analyses in Figure 2-3 Download Table 2-3, DOCX file.

10.1523/ENEURO.0380-25.2026.t2-4 Table 2-4 Statistical output for AUC GCaMP fiber photometry data in Figure 2-3 Download Table 2-4, DOCX file.

**Table 3.** Statistical output for AUC GCaMP fiber photometry data in Figure 2

Expt phase	Measure	Comparison	*T* value	*p* value	Significant?	Figure
SA	AUC (*n* = 11)	SA1 vs SA4	*t*_(10)_ = 5.143	0.0004	***	2*A*, right
Extinction	AUC (*n* = 10)	Ext1 vs Ext6	*t*_(9)_ = 3.000	0.0150	*	2*B*, right
Extinction/reinstatement	AUC (*n* = 10)	Ext6 vs Cue	*t*_(9)_ = 3.413	0.0077	**	2*C*, right
Extinction/reinstatement	AUC (*n* = 9)	Ext6 vs Pellet + cue	*t*_(8)_ = 2.393	0.0436	*	2*D*, right

10.1523/ENEURO.0380-25.2026.t3-1 Table 3-1 Statistical output for bootstrapping analyses in Figure 3-2 Download Table 3-1, DOCX file.

10.1523/ENEURO.0380-25.2026.t3-2 Table 3-2 Statistical output for AUC GRAB_DA fiber photometry data in Figure 3-2. Download Table 3-2, DOCX file.

10.1523/ENEURO.0380-25.2026.t3-3 Table 3-3 Statistical output for bootstrapping analyses in Figure 3-3 Download Table 3-3, DOCX file.

10.1523/ENEURO.0380-25.2026.t3-4 Table 3-4 Statistical output for bootstrapping analyses in Figure 3-4 Download Table 3-4, DOCX file.

10.1523/ENEURO.0380-25.2026.t3-5 Table 3-5 Statistical output for AUC GRAB_DA fiber photometry data in Figure 3-4. Download Table 3-5, DOCX file.

A different pattern emerged for DA traces (Fig. 3; full model output in Tables 4, 5). Upon lever press, we observed a small increase in DA in SA1 that appeared to become more robust and shift earlier in time by SA4 (Fig. 3*A*). During SA4, this DA peak was followed by a persistent dip below baseline (Fig. 3*A*, left). These differences between SA1 and SA4 were corroborated by bootstrapping analysis (Fig. 3*A*, left). In addition, we analyzed AUC for the 0–1 s window to attempt to capture the reduction in DA signal following the lever press as it shifted earlier in time (i.e., out of this window; Fig. 3*A*, right) but only a trend was found (*p* = 0.09).

**Table 4.** Statistical output for bootstrapping analyses in Figure 3

Expt phase	Measure	Factors in analysis	Time 95% CI ≠ 0	Significantly different?	Figure
SA	GRAB_DA response to lever press, *z*-scored trace (*n* = 11)	Bootstrapping		−2.01 to −1.07 s, 0.285–1.53 s, 1.79–5.23 s, 5.56–6.83 s, 8.55–10.0 s	3*A*, left
		SA1	1.79–3.55 s		
		SA4	−5.00 to −1.41 s, −1.13 to 1.77 s, 2.38–10.0 s		
Extinction	GRAB_DA response to lever press, *z*-scored trace (*n* = 11)	Bootstrapping		n.s.	3*B*, left
		Ext1	0.697–7.47 s, 7.79–9.77 s		
		Ext6	n.s.		
Extinction/reinstatement	GRAB_DA response to lever press, *z*-scored trace (*n* = 11)	Bootstrapping		n.s.	3*C*, left
		Ext6	n.s.		
		Cue test	n.s.		
Extinction/reinstatement	GRAB_DA response to lever press, *z*-scored trace (*n* = 11)	Bootstrapping		n.s.	3*D*, left
		Ext6	n.s.		
		Pellet + cue test	n.s.		

**Table 5.** Statistical output for AUC GRAB_DA fiber photometry data in Figure 3

Expt phase	Measure	Comparison	*T* value	*p* value	Significant?	Figure
SA	AUC (*n* = 11)	SA1 vs SA4	*t*_(10)_ = 1.970	0.0856	n.s.	3*A*, right
Extinction	AUC (*n* = 10)	Ext1 vs Ext6	*t*_(9)_ = 0.4708	0.6490	n.s.	3*B*, right
Extinction/reinstatement	AUC (*n* = 10)	Ext6 vs Cue	*t*_(9)_ = 2.589	0.0293	*	3*C*, right
Extinction/reinstatement	AUC (*n* = 10)	Ext6 vs Pellet + cue	*t*_(9)_ = 2.519	0.0359	*	3*D*, right

For consistency, the same AUC time windows used for analysis of GCaMP and GRAB_DA signals during the SA phase of the experiment (0–3 s and 0–1 s, respectively) were used for analysis of these signals in all animals during extinction and reinstatement phases (see next sections). However, it should be noted that no single window was optimal to capture temporally shifting DA responses across the experiment.

Overall, fiber photometry results obtained during SA training suggest that MSN activation state, as reflected by calcium transients, decreases from baseline levels upon a lever press that results in food reward and that this develops over the course of training. In contrast, DA release associated with active responding rises from baseline levels, and this peak becomes more robust and moves to precede the lever press. A potential explanation is that DA release begins to track the lever entering the chamber (which precedes the lever press) as the rats learn to perform the task (see below, Analyses of relationships between photometry signals and task-related behavior other than lever pressing).

### Photometry analysis of extinction sessions

We performed the same analysis described above for the first and last sessions (Ext1 and Ext6) of extinction training. For GCaMP, bootstrapping analysis indicated a small post-press decrease during Ext1 that is eliminated by Ext6 (Fig. 2*B*). When comparing the AUC for these two sessions with a paired *t* test, this change is significant (Fig. 2*B*, right; Table 3; *t*_(9)_ = 3.000, *p* = 0.0150). In fact, there was very little GCaMP response at any point in Ext6. Of note, one rat made no responses during Ext6 and therefore could not be included in analysis. For DA, bootstrapping analysis showed a small positive peak preceding the lever press on Ext1, similar to what was observed during SA4 (and thus perhaps reflecting anticipation of reward), but this was not evident during Ext6 (Fig. 3*B*, left). Analysis of AUC during the window of the positive peak (0–1 s, 0 being the lever press) did not detect any peak in either Ext1 or Ext6 with no significant difference between the two sessions (Fig. 3*B*, right), likely because the timing of peak onset and offset differed for individual animals, making it difficult to select an ideal window for AUC analysis. Bootstrapping also indicated a post-press dip below baseline during Ext1 which was dissipated by Ext6 (Fig. 3*B*, left). Together, these findings indicate a loss of DA responding over the course of extinction training. Furthermore, these data suggest that the calcium and DA responses observed in early extinction are not due to the motor action of the lever press (as the motor action is equivalent across extinction sessions) but rather due to the reward and learned operant response outcome. However, when comparing these two sessions it is important to keep in mind that the number of trials completed in Ext6 is greatly reduced compared with Ext1, which could potentially have affected our analysis. This and similar issues are addressed by additional analyses presented later in Results.

### Photometry analysis during reinstatement tests

Reinstatement tests allowed us to further examine NAcc MSN calcium and DA responses in a reward-seeking context. When examining MSN calcium via GCaMP, bootstrapping analysis indicated a small increase from baseline that overlapped with the light cue presentation in the cue-primed (Fig. 2*C*, left) and pellet + cue-primed tests (Fig. 2*D*, left). When assessed with AUC, this increase in calcium was significant for both reinstatement tests compared with the last extinction session (paired *t* tests; cue: *t*_(9)_ = 3.413, *p* = 0.0077; pellet + cue: *t*_(8)_ = 2.393, *p* = 0.0436) (Fig. 2*C*, right, *D*, right). For DA transients, there was an apparent increase for cue-primed reinstatement and pellet-primed reinstatement (positive peak overlapping with cue presentation) (Fig. 3*C*, left, *D*, left). While neither was considered a likely peak by bootstrapping analysis (Fig. 3*C*, left, *D*, left), analysis with AUC indicated a significant increase in magnitude of the DA response during both reinstatement tests compared with the last extinction session (*t*_(*9*)_ = 2.589, *p* = 0.0293 and *t*_(*8*)_ = 2.519*, p* = 0.0359 for cue- and pellet + cue-primed reinstatement, respectively; Fig. 3*C*, right, *D*, right).

### Photometry signals during first lever press, first five lever presses, and first 10 min of experimental sessions

As noted above in Photometry analysis of extinction sessions, sessions may differ in the number of trials completed, which can affect analysis of photometry data. Therefore, for both GCaMP (Extended Data Fig. 2-2, Extended Data Table 2-2) and GRAB_DA signals (Extended Data Fig. 3-3, Extended Data Table 3-3), we analyzed data during the first lever press of the session, the first five lever presses of the session, and the first 10 min of the session for key sessions across all experimental phases (SA1, SA4, Ext1, Ext6, Cue, Pellet + cue). We included analysis of the first 10 min because this is when most rats begin responding (even though they may differ in lever press number during this period), enabling us to assess the pattern when time is held constant. For GCaMP traces, we found that there was little change from baseline in the first lever press across all sessions, but when we examined binned data for the first five lever presses or the first 10 min, we saw traces of similar shape compared with the average for the full session (Extended Data Fig. 2-2). For GRAB_DA, across experimental phases, we observed traces of similar shape for the first lever press, the first five lever presses, the first 10 min, and the full session, although similarity to the full session is greatest for the first five presses and first 10 min (compare Extended Data Fig. 3-3, Fig. 3). Furthermore, the maximum amplitude of the response to the first lever press is notably larger for all sessions when compared with the traces for the first five lever presses and the first 10 min (Extended Data Fig. 3-3; note different *y*-axis for first lever press data). These data suggest that GRAB_DA and GCaMP responses are largely consistent across a recording session, with some changes in maximum amplitude. However, it is worth noting that the first lever press for GCaMP and GRAB_DA do differ in that only the GRAB_DA traces are similar to the average traces for the full session. This potentially suggests that DA tracks with the onset of the availability of reward (onset of session) while calcium may track with the cumulative experience rather than each reward.

### Relationships between GCaMP and GRAB_DA signals

**Figure 5.**
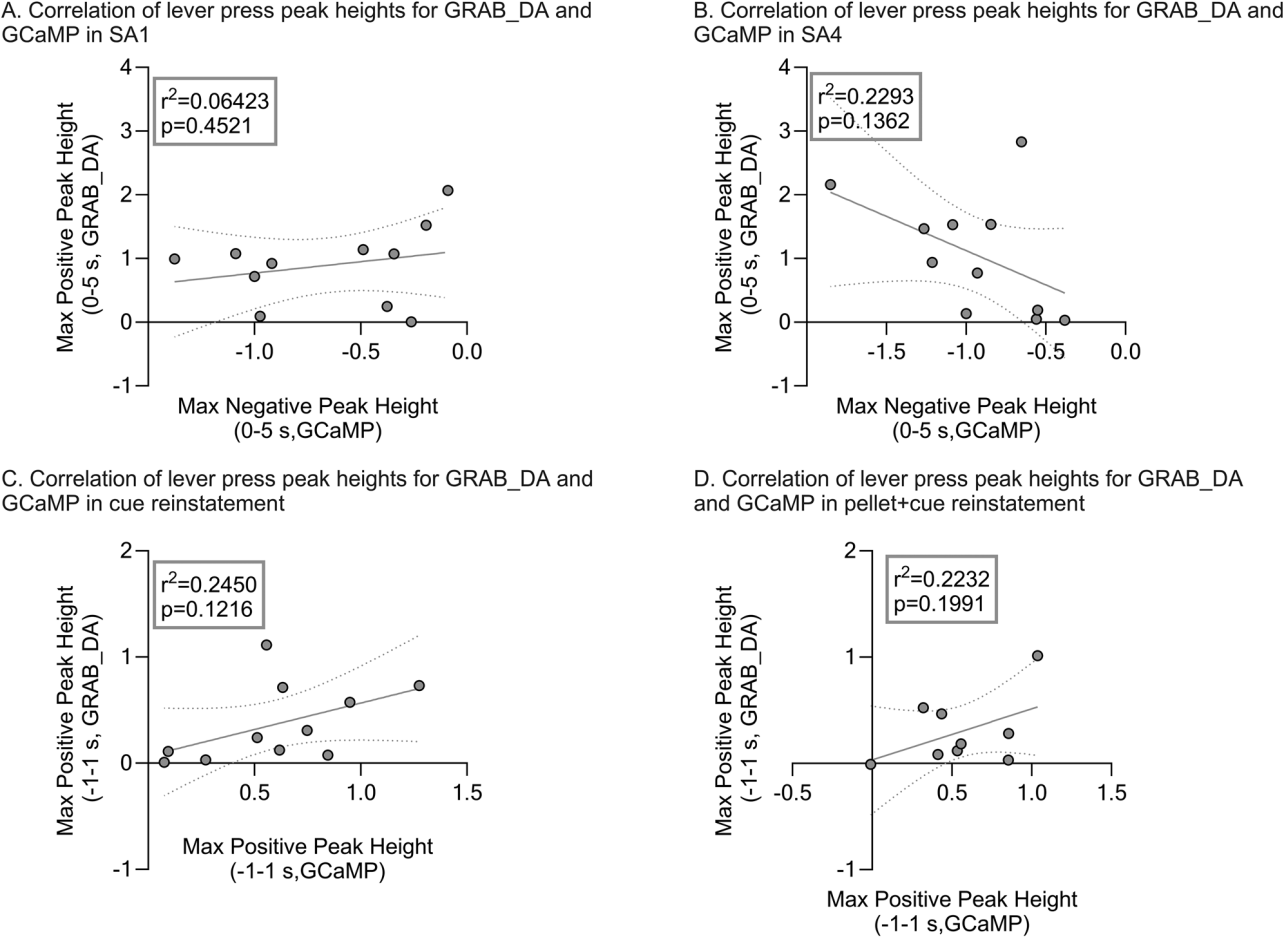
Simple linear regression analysis assessing the relationship between the maximum peak heights for GRAB_DA and GCaMP traces for individual rats across key behavioral sessions. Maximum peak height, positive or negative, was determined for each lever press in the session and the average maximum peak was determined for each rat in the time bins listed in the *y*-axes of the graphs (selected to correspond to Figs. 2, 3). Full traces are shown in Figure 4. The linear regression line is shown in black on all graphs with the 95% confidence intervals indicated by dashed lines. *r*^2^ and *p* values for the regression analysis are provided within each graph. Dots indicate individual rats. ***A***, Correlation between maximum peak height values for GRAB_DA and GCaMP during SA1. ***B–D***, Same as analysis as described in ***A*** but for SA4 (***B***), cue reinstatement (***C***), and pellet + cue reinstatement (***D***).

Simultaneously measuring both GCaMP and GRAB_DA signals in the same animal offers the opportunity to examine their relationship, albeit with caveats described in Materials and Methods, Methodological considerations for sensor multiplexing. Figure 4 shows GCaMP and GRAB_DA traces superimposed on each other for key experimental sessions. Visually, this reveals robust synchrony between the timing of the two signals (Table 6 shows statistical ouput). Next we explored whether magnitudes of the signals were correlated for individual rats (Fig. 5). For the SA phase, we decided to focus on the most prominent features of each signal—the post-press GCaMP dip (negative peak) and the peri-press GRAB_DA response (positive peak). For each, we determined the maximal amplitude attained between 0 and 5 s, selecting this broad window to accommodate individual differences in peak timing. While no significant correlations were observed, there was a trend (*p* = 0.136) toward a correlation on SA4, i.e., rats with a greater maximum negative peak height for GCaMP showed a greater maximum positive peak height for GRAB_DA when examining whole session data (Fig. 5*B*). Analysis at the level of experimental groups indicated that the magnitude of both the GCaMP dip and the GRAB_DA peak tended to increase over SA sessions 1–4 (Figs. 2, 3); individual analyses in Figure 5 suggest a potential relationship between the two changes. Limited or lack of detectable signal in Ext sessions precluded a similar analysis. However, for the reinstatement tests, we again designed the analysis to focus on the most prominent feature of each signal, namely, the positive peaks associated with the lever press for both signals. We observed trends toward a correlation between the maximum positive peak heights for GCaMP and GRAB_DA during both the cue (*p* = 0.121) and the pellet + cue (*p* = 0.199) reinstatement tests, again supporting a possible relationship between the two signals (Fig. 5*C*,*D*).

**Table 6.** Statistical output for bootstrapping analyses in Figure 4

Expt phase	Measure	Factors in analysis	Time 95% CI ≠ 0	Significantly different?	Figure
SA	GRAB_DA or GCaMP SA1 response to lever press, *z*-scored trace (*n* = 11)	Bootstrapping		2.55–5.12 s	4*A*, left
		GCaMP	8.10–9.01 s		
		GRAB_DA	−1.84 to −0.499 s, 1.80–3.52 s		
SA	GRAB_DA or GCaMP SA4 response to lever press, *z*-scored trace (*n* = 11)	Bootstrapping		−2.74 to −1.58 s, −0.436 to 3.57 s	4*A*, right
		GCaMP	0.477–6.69 s		
		GRAB_DA	−4.49 to −1.40 s, −1.17 to 0.430 s, 2.33–9.98 s		
Extinction	GRAB_DA or GCaMP Ext1 response to lever press, *z*-scored trace (*n* = 11)	Bootstrapping		n.s.	4*B*, left
		GCaMP	0.360–1.83 s		
		GRAB_DA	0.726–7.34 s, 7.77–9.73 s		
Extinction	GRAB_DA or GCaMP Ext6 response to lever press, *z*-scored trace (*n* = 10)	Bootstrapping		n.s.	4*B*, right
		GCaMP	n.s.		
		GRAB_DA	n.s.		
Reinstatement	GRAB_DA or GCaMP Cue response to lever press, *z*-scored trace (*n* = 11)	Bootstrapping		n.s.	4*C*, left
		GCaMP	−0.214 to 4.46 s		
		GRAB_DA	n.s.		
Reinstatement	GRAB_DA or GCaMP Pellet + cue response to lever press, *z*-scored trace (*n* = 10)	Bootstrapping		n.s.	4*C*, right
		GCaMP	−0.014 to 2.09 s		
		GRAB_DA	n.s.		

### Relationships between photometry signals and lever pressing for individual rats

**Figure 6.**
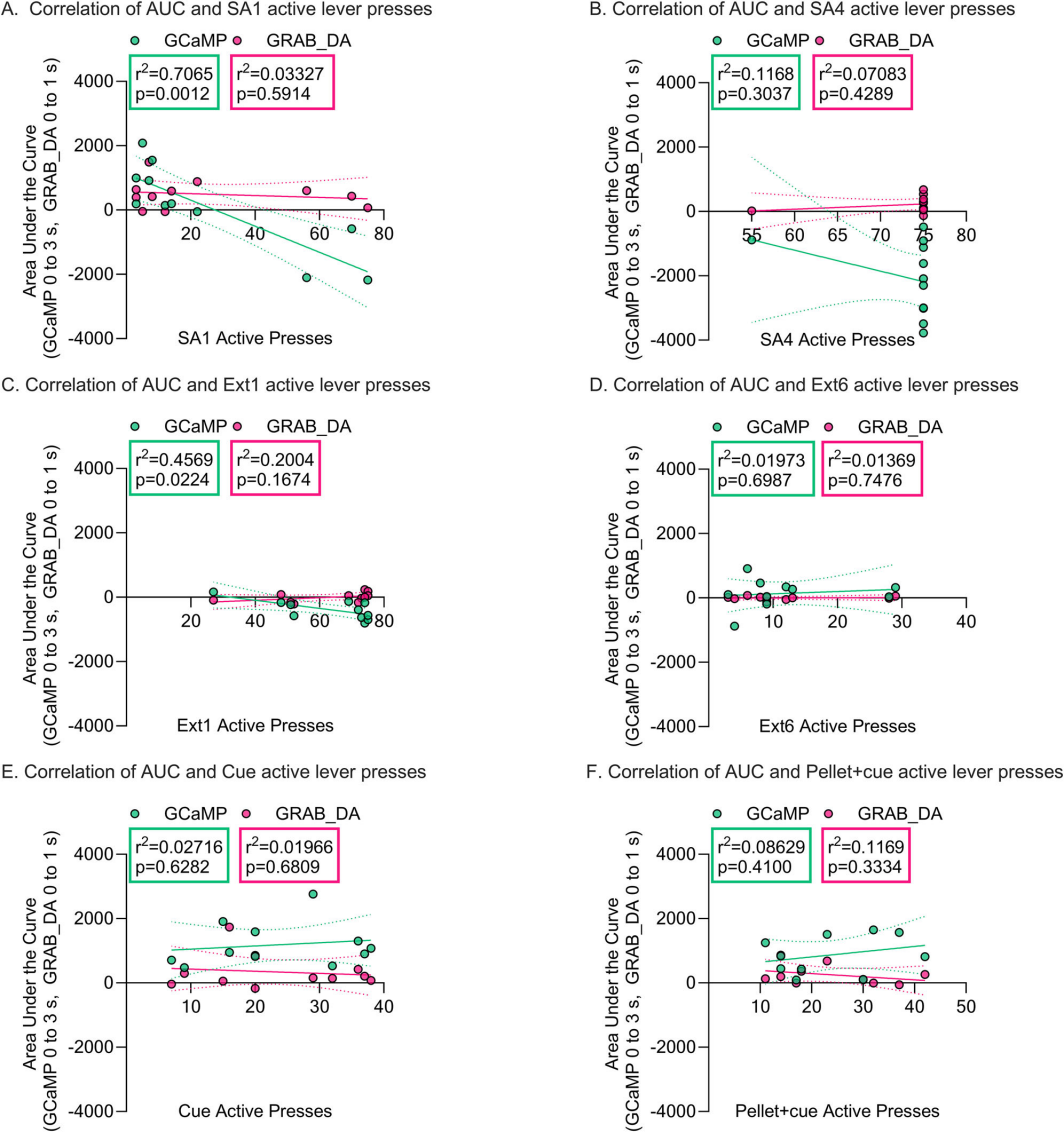
Simple linear regression analysis assessing the relationship between AUC of GRAB_DA or GCaMP traces and lever presses across behavioral sessions. AUC was determined for each lever press in the session and averaged for each rat in the time bins listed in the *y*-axes of the graphs (selected to correspond to time bins in Figs. 2, 3). Full traces and AUC values are shown in Figure 2 (GCaMP) and Figure 3 (GRAB_DA). The linear regression line is shown in green for GCaMP and magenta for GRAB_DA on all graphs with the 95% confidence intervals indicated by dashed lines. *r*^2^ and *p* values for the regression analysis are provided within each graph and color coded to their respective regression lines. Dots indicate individual rats. ***A***, Correlations for GCaMP and GRAB_DA AUC values versus active lever presses. ***B–D***, Same as analysis described in ***A*** but for SA4 (***B***), extinction (Ext) 1 (***C***), Ext6 (***D***), cue reinstatement (***E***), and pellet + cue reinstatement (***F***).

To evaluate the relationship between photometry signals and lever presses for individual animals, we tested correlations between AUC data for GCaMP or GRAB_DA AUC (0–3 and 0–1 s windows, respectively, as in Figs. 2, 3) and lever presses on SA1, SA4, Ext1, Ext6, Cue test, and Pellet + Cue test (Fig. 6). No significant correlations were found for GRAB-DA AUC data. However, we found a significant correlation on both SA1 (*p* = 0.001) and Ext1 (*p* = 0.022) between the number of lever presses and the magnitude of the GCaMP dip (negative AUC). The increase in the magnitude of the GcAMP dip from SA1 to SA4 as lever pressing and trial completion also increase (Fig. 2, Extended Data Fig. 2-1) suggests that a larger GCaMP dip is related to increased responding. From this perspective, it makes sense that rats with higher lever pressing on SA1 have a larger dip and that rats who continue pressing on Ext1 also have a larger dip compared with rats that responded less overall. Please note that no significant correlation was expected for GCaMP (or GRAB_DA) on SA4 because all but one rat completed all 75 trials, eliminating lever press variability. Absence of other significant correlations is not unexpected given individual variability of our measures. For example, AUC was determined for the same period of time for all rats, while the actual timing of positive and negative peaks varied by rat. It is possible that some correlations would have strengthened with additional rats.

We analyzed DA and calcium signals time-locked to lever presses and therefore interpreted these signals in the context of operant responding. However, additional behaviors unrelated to the operant task during photometry sessions could influence lever pressing and perhaps contribute to photometry signals, such as licking or biting the lever or magazine. To assess this, we evaluated videos made during photometry recordings. In general, we did not see evidence of licking or biting behaviors, supporting the relationship of our observed photometry signals to lever pressing. However, the position of the camera in relation to the operant chamber made viewing the lever while the animal was in front of it difficult. Therefore we cannot report a quantitative assessment of physical interactions with the lever that were not a lever press.

### Analyses of relationships between photometry signals and task-related behavior other than lever pressing

The bulk of the data described above concerns photometry signals time-locked with lever pressing. To gain insight into their potential significance for other aspects of the task, we began by analyzing photometry traces for all experimental phases time-locked to lever entry. The rationale, presented above in the section titled Photometry analysis of SA sessions, is that the positive GRAB_DA peak associated with active lever pressing becomes more robust from SA1 to SA4 and moves to precede the lever press. This could be explained if DA release begins to track the lever entering the chamber (which precedes the lever press). In fact, when we analyzed GRAB_DA traces time-locked to lever entry, we observed a larger and sharper positive peak at ∼*t* = 0 on SA4 compared with SA1, possibly suggesting that lever entry acquires motivational significance as the task is learned, whereas this peak appeared to decline from Ext1 to Ext6 (Extended Data Fig. 3-4; Extended Data Tables 3-4, 3-5). When we performed the same analysis for GCaMP, the initial peak (∼*t* = 0) was better aligned between SA1 and SA4 when time-locked to lever entry (Extended Data Fig. 2-3; Extended Data Tables 2-3, 2-4) than when time-locked to active lever presses (Fig. 2), perhaps suggesting a relationship to response preparation. While not significant, its relative amplitude appeared greater on Ext1 versus Ext 6 when time-locked to lever entry (Extended Data Fig. 2-3) whereas this was reversed when time-locked to lever presses (Fig. 2). Both DA and calcium responses time-locked to lever entry during reinstatement tests were very small but were statistically different from Ext6 when AUC were analyzed (Extended Data Figs. 2-3, 3-4; Extended Data Tables 2-4, 3-5).

Next, we determined the latency of responding (i.e., the delay between lever insertion and lever press) across experimental phases and tested relationships between latency and photometry signals across experimental phases. We found that the latency of active lever presses decreased from SA1 to SA4, increased during extinction training, and remained relatively high during reinstatement tests (Extended Data Fig. 2-4*A*). Only one significant correlation was observed when we tested the relationship between latency and GCaMP or GRAB_DA photometry signals (quantified as AUC; Extended Data Fig. 2-4*B–G*), namely, an inverse relationship between the GCaMP AUC and latency during Ext1 (*p* = 0.0373; Extended Data Fig. 2-4*D*). A similar relationship was trending toward significance (*p* = 0.0571) in SA1. Thus, when the latency of responding during SA1 or Ext1 is shorter (i.e., more like SA4), then the magnitude of the photometry response (dip below baseline) is greater (i.e., more like the averaged SA4 trace). This could indicate a relationship between Ca^2+^ signaling and the vigor of responding.

## Discussion

Prior studies have demonstrated compatibility of red-shifted DA sensors with GCaMP in nonoperant behavioral models (Patriarchi et al., 2018, 2020). Here we used this approach to assess the relationship between NAcc MSN activation state (using calcium as a proxy; see next section) and DA levels during food self-administration, extinction, and reinstatement. We focused on signals time-locked to lever presses although other aspects of the task were also explored. Our finding of a large dip in the *z*-scored GCaMP signal during food self-administration agrees with prior observations of decreased NAc MSN neuronal firing rate during food consumption (see below). This dip, which is not significant for “free” pellet consumption during magazine training, develops over the course of SA training. It is also observed in the first extinction session, although it disappears over subsequent extinction sessions. These observations suggest this dip may be related to task learning and not solely food consumption. In the reinstatement phase, an elevation of MSN calcium parallels increased cue-induced responding. In the same rats, during self-administration training we observe a positive peri-press DA peak that initially follows lever pressing but moves earlier in time and appears to increase in size as training progresses. Our analysis of traces time-locked to lever entry shows that this DA peak moves to track this important predictive event. A post-press decrease following the initial positive peak in the DA signal also develops over training. Both the initial positive peak and the post-press decrease in DA are reduced in the first extinction session and eventually disappear with extinction learning, although the positive peri-press DA response is restored during reinstatement. We evaluated correlations between prominent features of calcium and DA signals for individual rats and found some interesting trends, supportive of a relationship between the two signals, but they did not reach statistical significance. However, we did observe significant correlations between GCaMP signals and both lever pressing and latency to lever press. Finally, in addition to analyzing full sessions across experimental phases, we assessed traces within sessions, binning by both time and response number, and largely did not see substantial within session changes in GCaMP or DA traces. Our study, the first to measure calcium and DA in the same animal on each day of an extinction-reinstatement task, adds to the field by revealing time-dependent changes in calcium and DA, sometimes in parallel and sometimes not, across phases of the task. With this foundation, the next step in future studies is to test the behavioral significance of calcium and DA signals and how the two may influence each other.

### Relationship between calcium levels and MSN activation

A number of publications mentioned below used electrophysiology to assess MSN activity, whereas we used a calcium sensor. This raises the important question of how to relate the two measures. A recent paper using a combination of electrophysiological recordings and calcium imaging concluded that fiber photometry calcium transients are poorly correlated with average MSN spiking (e.g., only 33% of photometry transients occurred within 500 ms of a burst) but better correlated with calcium in MSN dendrites (derived by measuring calcium with a GRIN lens while masking the somatic compartment; Legaria et al., 2022). Calcium in dendrites is very relevant to our over-arching goal of understanding the interplay between MSN activation, DA signaling, and motivated behavior. MSNs in vivo have bistable resting potentials, existing in a hyperpolarized down state and a depolarized up state from which they can fire action potentials (O’Donnell, 2003). L-type calcium channels are implicated in transitioning to the up state (Vergara et al., 2003; Tseng et al., 2007), so an increase in MSN calcium levels may be correlated with increased likelihood to respond to excitatory inputs even if a rise in this calcium level does not necessarily predict a spike. For this reason, we refer to calcium signals as a proxy for MSN activation state and cautiously interpret them as related to prior electrophysiological measures in food seeking tasks.

### Role of NAcc MSN and DA transmission in food consumption

As noted above, the role of NAcc MSN activity in food intake has been previously investigated using in vivo electrophysiology. This work found that a subset of neurons increase their firing rate during operant responding for food but exhibit a pause in firing during consumption that is necessary for consummatory behavior (Carelli et al., 2000; Nicola et al., 2004; Roitman et al., 2005; Frazier and Mrejeru, 2010; Krause et al., 2010). Although we acknowledge that calcium is only a proxy for neuronal activity, our calcium data seem to parallel this prior work, with a negative transient occurring after the lever press. This could be partially due to reward consumption, but there is also likely a learning component. Thus, if the observed calcium decrease was simply due to reward consumption, it would be similar when rats consume a “free” pellet during magazine training, which was not observed. Furthermore, it likely would be unchanged across SA sessions. Instead, we find that the decrease becomes more pronounced as self-administration training progresses. This could reflect higher trial completion and therefore a higher number of rewards eaten, but as we only analyzed completed trials, we believe this to be unlikely. Supporting a role for learning, a study observing a pause during consumption found that this pause was modulated depending on the presentation of a predictive cue (Nicola et al., 2004). The lever extension at the start of the trial likely acts as our predictive cue or “DS” and could be modulating the decrease in calcium signaling as the task is learned. Supporting this, a small decrease from baseline upon lever press remains evident in Ext1. In these extinction sessions, there is zero consumption, indicating that some portion of this decrease is due to other aspects of behavior. Future studies manipulating NAcc function will be required to isolate the behavioral component encoded by this decrease from basal calcium.

DA has long been thought to be essential for reward learning, but the exact nature of its role is debated. The classic view of DA is that it encodes discrepancies between the prediction of reward as a result of behavior and the actual consequences, termed “reward-prediction error” (Keiflin and Janak, 2015). In this model of DA as a learning signal, it would be expected that DA responses would be elevated early in food self-administration training (when the rat is learning the relationship between a lever press and reward delivery), decrease later in training (as the reward becomes more predicted), and increase in extinction (when the response-outcome contingency changes again). That pattern of DA responding is not reflected in our data. Since this difference could be due to our analysis of session averages, we also examined the DA response to the first lever press, the first five lever presses, and lever presses within the first 10 min and found that the DA signal during these periods was similar to the whole session averages. A more recent theory of DA's significance proposes that it acts as a value signal and, particularly in the NAc, high DA indicates that effortful work will be beneficial (Berke, 2018). This tracks better with our observations, with DA increasing as the rat completes more trials and becomes more assured of the value of the lever press and decreasing in Ext1 when the value of that work reduces. Other theories propose DA as a signal of perceived saliency (Kutlu et al., 2021) or a tool for retrospective learning (Jeong et al., 2022), both of which would also track with our observations of DA transients during food SA and extinction.

### Role of NAcc MSN and DA transmission in food seeking

The reinstatement phase isolates the value of the reward-associated cue and its ability to invigorate behavior. We found significantly increased calcium transients in both the cue-primed and pellet + cue-primed reinstatement tests compared with the last session of extinction. Likewise, there was an increased DA response during both reinstatement tests compared with the last session of extinction. Thus, calcium and DA exhibit very different responses during self-administration (above) but track together during reinstatement tests. Some studies design their task to investigate both the cue in isolation and the reward response by having the cue precede the operant action. These studies found increased activity of NAc neurons during the cue period which was facilitated by DA (Yun et al., 2004; du Hoffmann and Nicola, 2014). Furthermore, intra-NAc DA receptor antagonism with SCH23390 or raclopride attenuated cue-induced food reinstatement (Guy et al., 2011). These results, along with ours, suggest that the ability of cues to promote food seeking depends upon MSN activation (presumably driven by glutamate) enhanced by neuromodulatory effects of DA.

### Role of D1 and D2 MSN in observed responses

The principal neurons of the NAcc are GABAergic MSNs that express either D1 or D2 DA receptors (D1 MSN, D2 MSN). D1 MSN have often been implicated in driving motivated behaviors whereas D2 MSN are less involved or oppose such behaviors, although current theories emphasize the importance of balance and interactions between these pathways (Burke et al., 2017; Bariselli et al., 2019; Allichon et al., 2021). We expect that both D1 and D2 MSN are represented in our population signal but may contribute differently to observed signals. This could be investigated by repeating our experimental paradigm with GCaMP selectively expressed in D1 versus D2 MSN. Several prior studies measured activity of D1 MSN and D2 MSN (using electrophysiology or fiber photometry) during palatable solution consumption. One study found that lateral habenula-projecting shell D1 MSN reduce activity to permit consumption while D2 MSN do not reliably change activity (O’Connor et al., 2015), while others observed D2 MSN inhibition associated with consumption (Guillaumin et al., 2023) or inhibition of both MSN subtypes after a lick but differences preceding the lick (Walle et al., 2024). Finally, in a DA/calcium multiplexing study with GCaMP6f expressed in D1 MSN, a peak in DA preceded a slow rise in calcium when rats were presented with unpredictable rewards (Patriarchi et al., 2020). The different results likely reflect substantially different paradigms and methodologies and, together with other analyses performed as part of these prior studies, suggest that a complex interplay of D1 and D2 MSN governs consummatory behavior.

### Relationship between DA and calcium signaling in prior multiplexing studies

The present study is the first to multiplex DA and calcium sensors in an operant task, and we found that the relationship between DA and calcium signals varied across phases of this task. This also holds when other behaviors are examined. In the prior multiplexing study in which both sensors were expressed in the NAc, both DA and calcium signals increased when mice consumed 5% sucrose, while calcium increased but DA decreased in response to unpredictable foot-shocks (Patriarchi et al., 2018). In an analysis of pavlovian conditioning in the same study in which only DA was measured, recordings in NAc revealed an increase in DA in response to predictive cues that became more pronounced upon repeated cue-reward pairings while the DA response associated with the US (sucrose) became smaller; during extinction, the DA response to the CS declined across sessions (Patriarchi et al., 2018). These data show both similarities and differences compared with our operant task. On the similar side, we observed that the DA peak associated with cue presentation was more robust on SA4 compared with SA1 (it also moved earlier in time) and this peak was eliminated during extinction training. In contrast, during the period of consumption in our SA sessions, DA levels were above baseline on SA1 but below baseline on SA4 (permutation testing on bootstrapped data confirmed a significant difference). It is also notable that the study of pavlovian conditioning found significant correlations between the amplitude of the DA response to the CS and the number of the session (for both learning and extinction) as well as a significant correlation between the DA response to the CS and the number of licks during the CS in learning and extinction sessions (Patriarchi et al., 2018), whereas we did not observe significant correlations between the GRAB_DA AUC and active lever presses during SA, extinction, or reinstatement. This could be due to our small sample size.

### Conclusions

In NAcc MSN, a decrease in calcium from baseline following the lever press became progressively more pronounced over the course of food self-administration training. Both a positive peri-press DA peak and a post-press DA dip also became more pronounced as training progressed. These responses were all reduced on the first day of extinction before disappearing (no detectable transients) by the last day of extinction. When measured during cue-primed and pellet + cue-primed reinstatement, significant increases in calcium and DA were observed relative to the last day of extinction. Our results provide new information about the interplay between calcium and DA in the NAcc across acquisition of food self-administration, extinction of this behavior, and reinstatement of operant responding.

Extended Data Figures and Tables can be found at https://doi.org/10.1523/ENEURO.0380-25.2026.

## Synthesis

Reviewing Editor: Tod E Kippin, University of California Santa Barbara Decisions are customarily a result of the Reviewing Editor and the peer reviewers coming together and discussing their recommendations until a consensus is reached. When revisions are invited, a fact-based synthesis statement explaining their decision and outlining what is needed to prepare a revision will be listed below. The following reviewer(s) agreed to reveal their identity: Jun Wang. Note: If this manuscript was transferred from JNeurosci and a decision was made to accept the manuscript without peer review, a synthesis may not be available.

I have now received two independent reviews of your manuscript and both emphasize the overall strength of the present manuscript but differ in terms the potential need for additional work. Namely, Reviewer 2 suggests a couple of additional analyses that may improve understanding of the relation between emission of behavioral responses and the changes in neuronal signaling. As such, I am recommending revision and resubmission. Please address all of the below comments in your resubmission. If you have any questions during the revision process then I am happy to consult with you.

Reviewer 1

This study investigates how dopamine release and neuronal calcium activity in the nucleus accumbens core evolve during the learning, extinction, and reinstatement of food-seeking behavior, using a dual-color fiber photometry approach that simultaneously records dopamine (rGRAB-DA3m) and MSN calcium (GCaMP8s) signals in the same rats. The authors show that during self-administration, dopamine transients shift from following to preceding lever presses as animals learn reward contingencies, while calcium signals exhibit a growing post-press decrease reflecting neuronal inhibition; both signals diminish with extinction and reappear during cue- or cue-plus-pellet-induced reinstatement, paralleling the recovery of motivated behavior. The work provides the first longitudinal demonstration of coordinated dopamine and calcium dynamics across all phases of an operant food-seeking task, revealing changing relationships between neuromodulator release and MSN activation state that mirror learning and relapse processes. Although the data are descriptive, the study establishes a technically rigorous foundation for future causal investigations of corticostriatal mechanisms underlying motivation and reward. I do not have concerns

Reviewer 2

The manuscript examines simultaneous dopamine and calcium dynamics in the nucleus accumbens core during acquisition, extinction, and reinstatement of food-seeking behavior using multiplexed fiber photometry. Overall, the authors demonstrate that dopamine and calcium signals display distinct temporal dynamics that evolve across phases of learning, extinction, and reinstatement. This provides a useful foundation for future mechanistic work. I particularly appreciate the authors' thorough and transparent discussion of the methodological caveats associated with multiplexed photometry. However, the functional interpretation of these physiological signals remains limited, largely due to insufficient behavioral characterization of the instrumental responses, as detailed below.

1.Relevant behavioral epochs.

Authors report GCamp or DA traces around lever-press.

In the behavioral procedure --with a trial structure-- another significant epoch is the start of the trial, i.e. the lever presentation. I wish authors also analyzed the photometry traces around this significant behavioral epoch. It would allow authors to compare trials where lever insertion is followed by a press versus those with longer latencies or no response. This could give a clearer picture of how motivation or response preparation is encoded.

Along the same lines, analyzing photometry signals during the consumption of the "free" pellet at the start of the last reinstatement session would allow to better dissociate signals evoked by appetitive operant responses vs consummatory behavior.

2. Response latency, and other behavioral measures

All lever presses are not alike. A likely difference between responses during reinforced sessions and late extinction is the latency to respond (the delay between lever insertion and lever press). Has this measure been recorded? If so, it would be important to report it and, if possible, test whether response latency correlates with the photometry signals. Such an analysis could provide useful insight into how dopamine and calcium activity relate to motivation or vigor of responding. Likewise the duration of magazine visits following lever press could provide valuable information to interpret differences in Ca2+ or DA signals.

3. Photometry signals during the first 10 min

To control for differences in trial numbers across sessions, the authors also report photometry signals for the first response in each session --which is a good approach. However, I'm puzzled by the additional analysis based on the first 10 minutes of each session. Unless the authors show that rats complete the same number of presses within this time window, this analysis likely reintroduces the same confound it was meant to avoid. A more appropriate comparison would be to average over a fixed number of responses (e.g., the first five lever presses).

Also, please clarify if it is the first nose-poke or lever press (Ls 453 and 457).

Minor:

Please specify the baseline epoch used for Z score nrmalization.

In the Recording parameters section (Lines 226-229), it's unclear whether the listed frequencies (330 Hz, 210 Hz, 450 Hz) refer to LED modulation frequencies used for lock-in demodulation or to sampling rates. Please clarify and specify the actual LED driving frequencies used for lock-in amplification.
