# Supplementary figures and images for: Dopamine and Calcium Dynamics in the Nucleus Accumbens Core during Food Seeking
Source: eNeuro. 2026 Apr 28;13(4):ENEURO.0380-25.2026. doi: 10.1523/ENEURO.0380-25.2026 (PMC13124030; doi:10.1523/ENEURO.0380-25.2026)

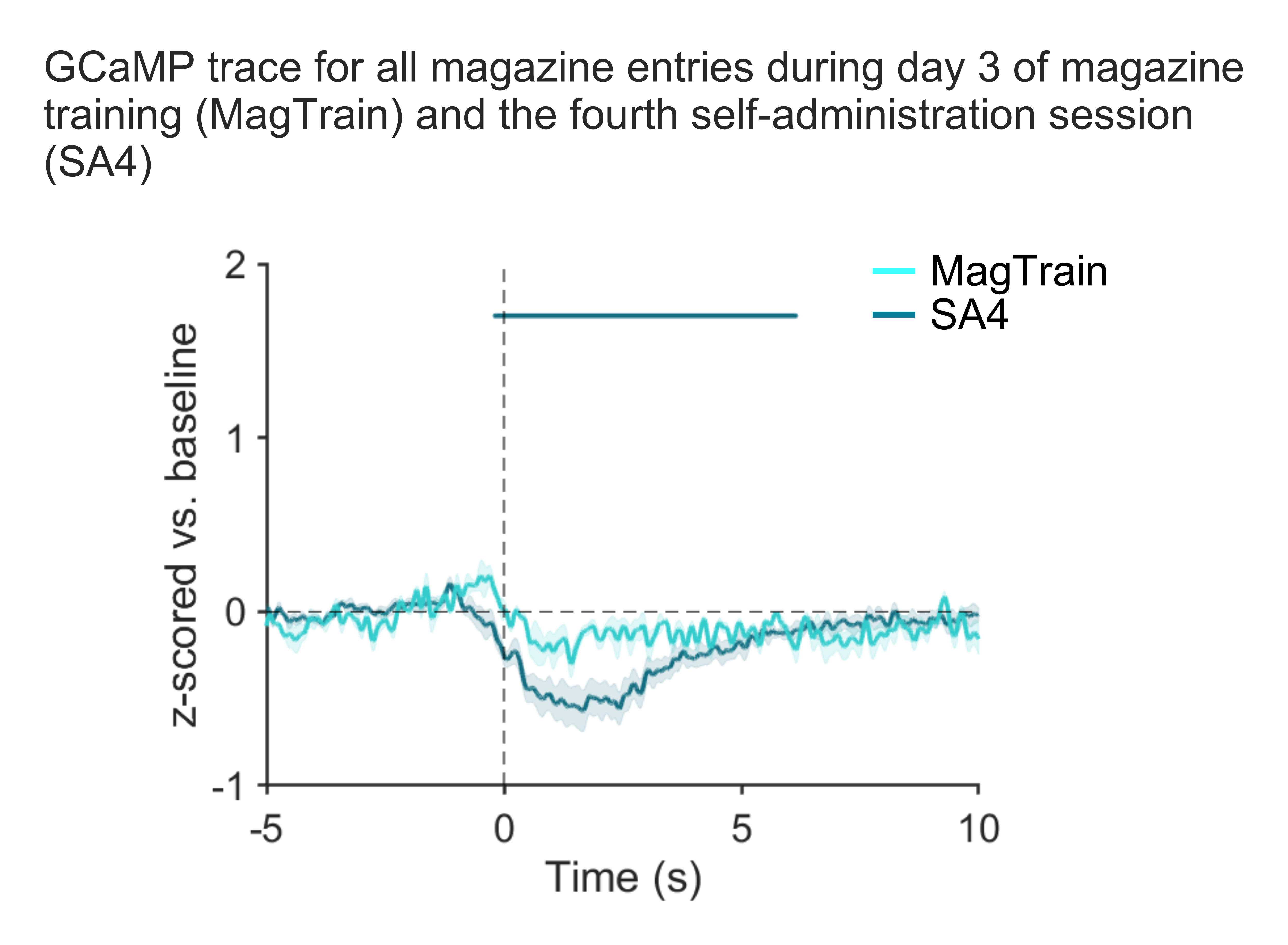

Supplement: Figure 2-1 — Fiber photometry recordings of GCaMP transients in the NAcc associated with “free” pellet consumption during magazine training. GCaMP z-scored mean traces time-locked to magazine entries in the third magazine training session (MagTrain) and the fourth self-administration session (SA4) normalized to a baseline period (-5 to 0 s). SEM is shown in shaded area around the mean. Black vertical dashed line at time 0 s indicates the magazine entry. The matching-colored line above the traces identifies a significant transient for SA4, i.e., a period in the 15-s window (-5 to 10 s) during which bootstrapping indicates 95% confidence that the mean is not equal to zero (baseline level). Full statistical output for experiments shown in this figure is presented in Table 2-1. Download Figure 2-1, TIF file. [file eneuro-13-ENEURO.0380-25.2026-s002.tif]

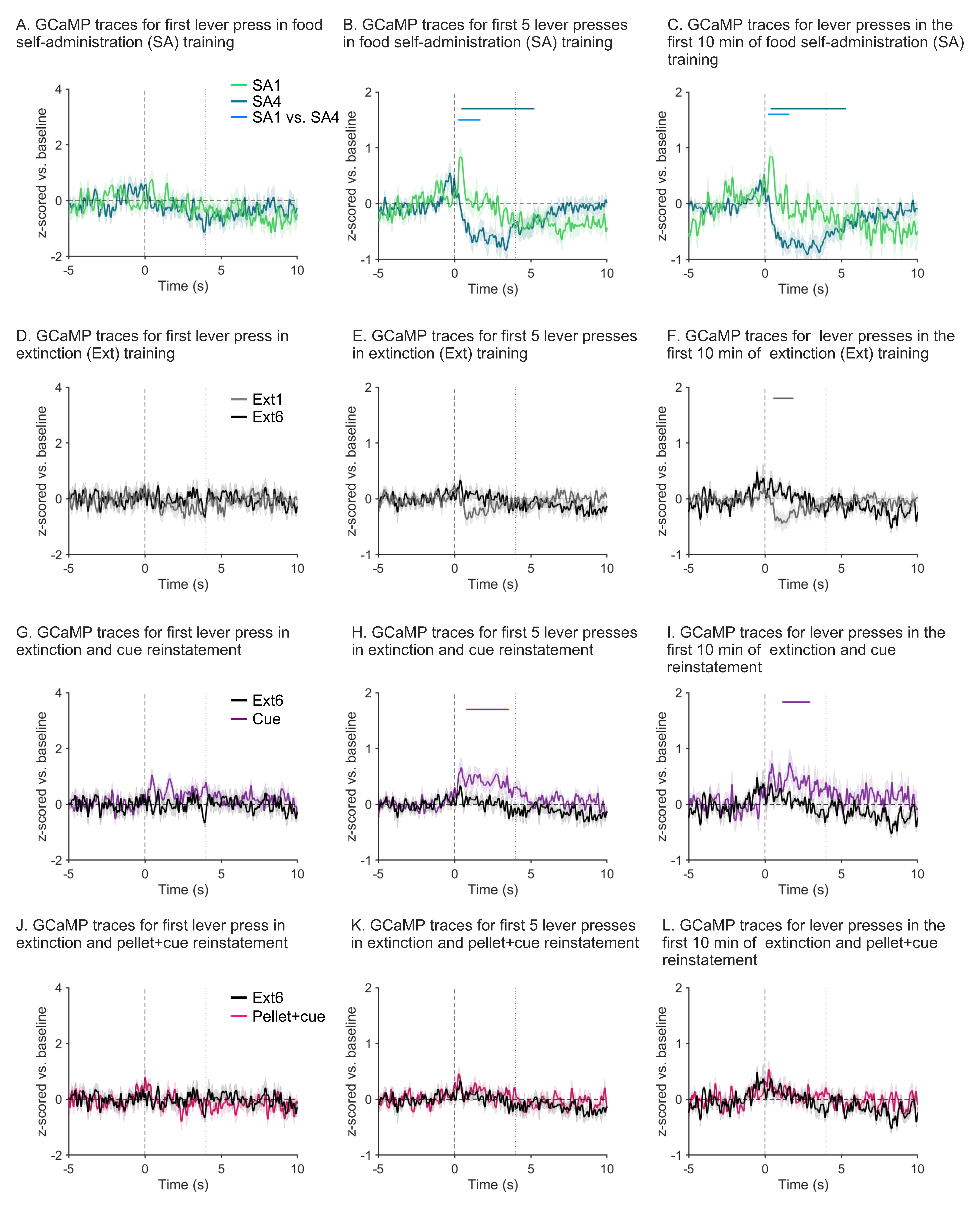

Supplement: Figure 2-2 — Fiber photometry recordings of NAcc GCaMP signals across food self-administration, extinction, and reinstatement sessions focusing on first lever press, first five lever presses, or the first 10 min of key sessions. A. z-scored mean GCaMP traces time-locked to the first active lever press in the first session of self-administration (SA1) and the last session of self-administration (SA4) normalized to a baseline period (-5 to 0 s). SEM is shown in shaded area around the mean. The black vertical dashed line at time 0 s indicates the lever press while the gray vertical dashed line at time 4 s indicates the end of the light cue. The matching-colored lines above the traces show periods in the 15-s window during which bootstrapping indicates 95% confidence that the mean is not equal to zero (baseline level). The light blue lines above the traces indicate time periods in which the bootstrapped confidence intervals are significantly different from each other. B. z-scored mean GCaMP traces time-locked to the first five active lever presses of SA1 and SA4. C. z-scored mean GCaMP traces time-locked to the active lever presses in first 10 min of SA1 and SA4. D-L. These panels show GCaMP traces, as described in A-C, during the first session of extinction (Ext1) and the last session of extinction (Ext6) (D-F), the last session of extinction (Ext6) and cue-primed reinstatement (Cue) (G-I), and the last session of extinction (Ext6) and pellet+cue-primed reinstatement (Pellet+cue) (J-L). Full statistical output for experiments shown in this figure is presented in Table 2-2. Download Figure 2-2, TIF file. [file eneuro-13-ENEURO.0380-25.2026-s009.tif]

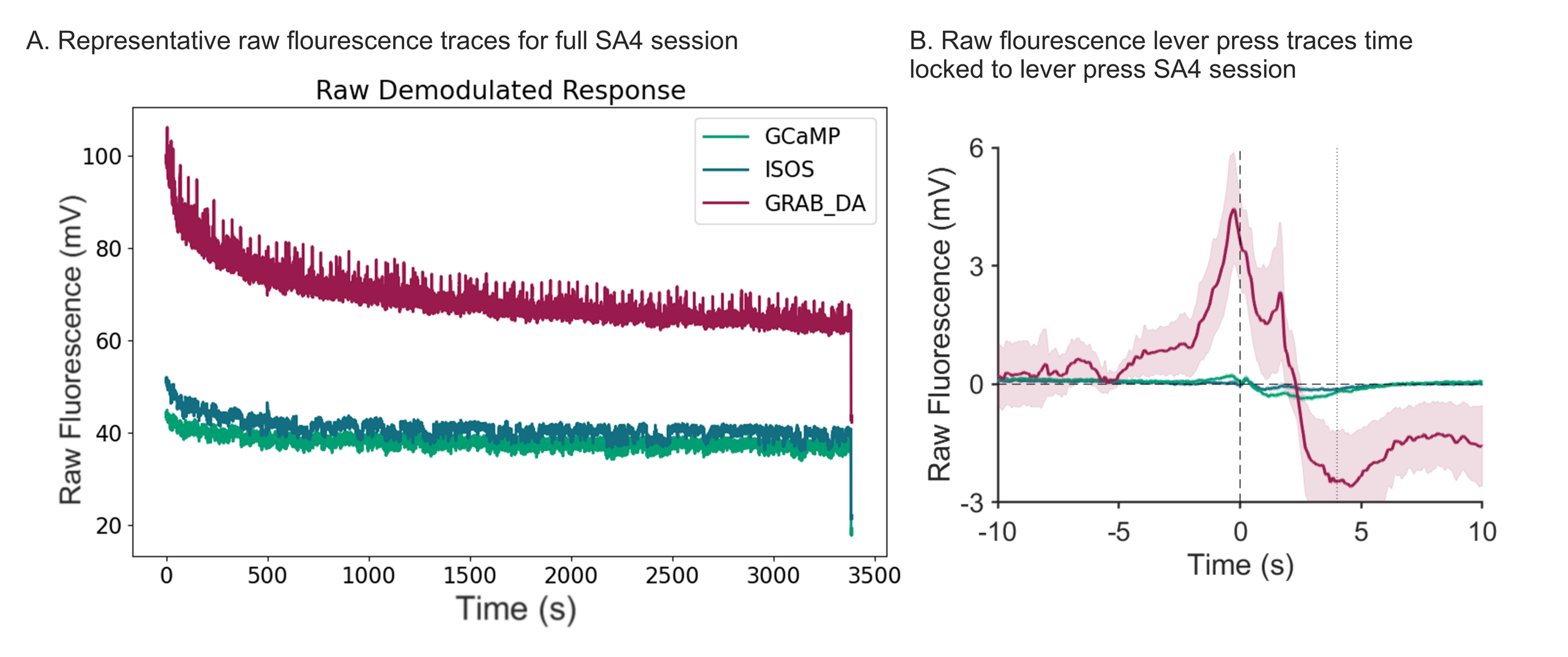

Supplement: Figure 3-1 — Raw fluorescence data for all recorded channels. A. Representative traces showing raw demodulated fluorescence data for SA4 of one rat. B. Raw fluorescence averaged across all rats (n = 11) for SA4 for 405 nm (isosbestic), 465 nm (GCaMP), and 560 nm (GRAB_DA) channels. SEM is shown as shaded area around the mean. Black vertical dashed line at time 0 s indicates the lever press. Gray vertical dashed line at time 4 s indicates the end of the light cue. ISOS, isosbestic. Download Figure 3-1, TIF file. [file eneuro-13-ENEURO.0380-25.2026-s005.tif]

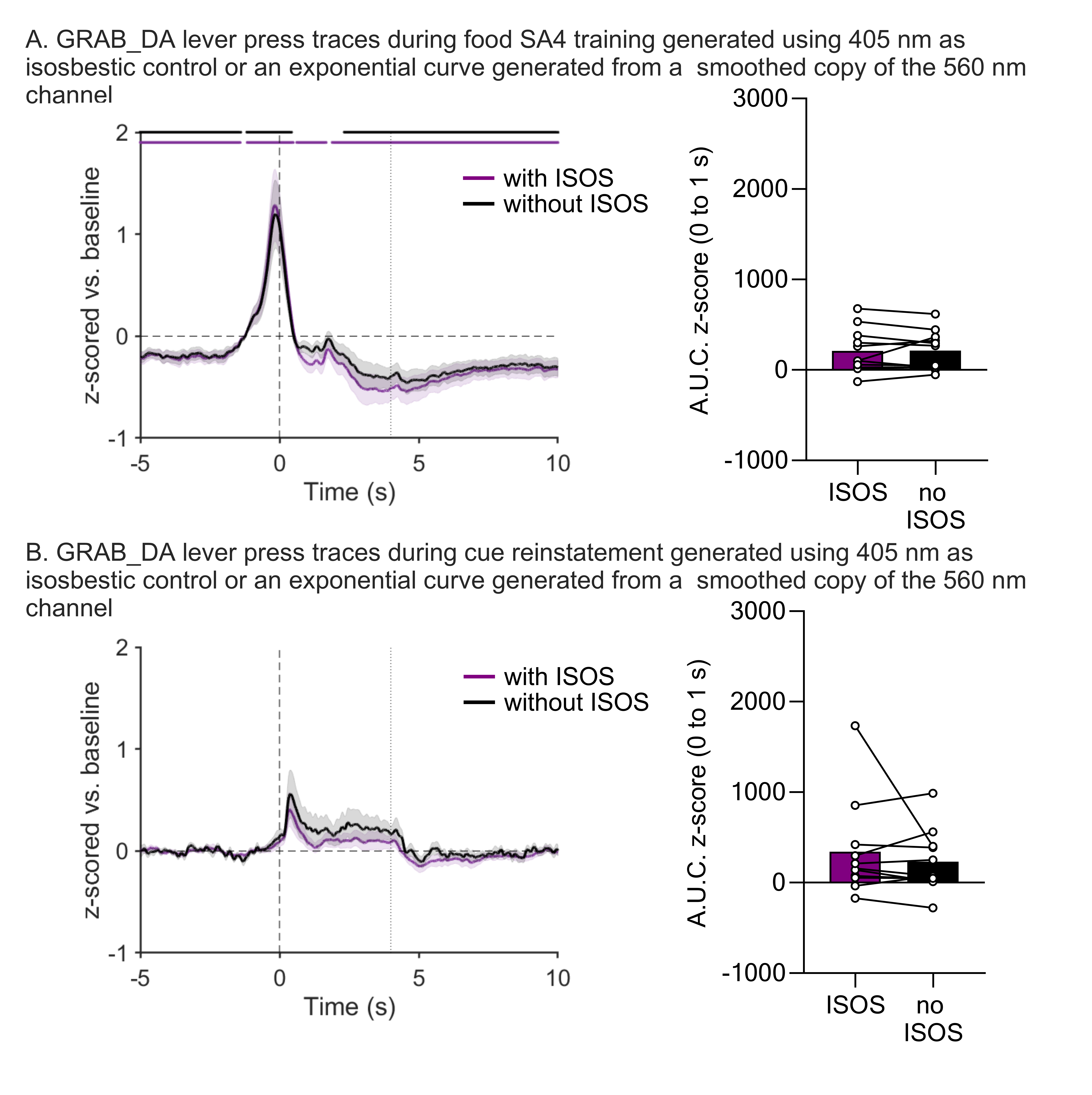

Supplement: Figure 3-2 — Fiber photometry recordings of GRAB_DA transients in the NAcc during food self-administration (SA4) analyzed with the inclusion of the 405 nm isosbestic channel and without. A. Left: z-scored mean DA traces time-locked to active lever presses in the last session (SA4) of self-administration normalized to a baseline period (-5 to 0 s). SEM is shown as shaded area around the mean. Black vertical dashed line at time 0 s indicates the lever press. Gray vertical dashed line at time 4 s indicates the end of the light cue. The matching-colored lines above the traces identify significant transients, i.e., periods in the 15-s window (-5 to 10 s) during which bootstrapping indicates 95% confidence that the mean is not equal to zero (baseline level). Right: Area under the curve for the traces shown in Left. Bars show mean (±SEM) for time window starting at the lever press (0 s) to 1 s after, while dots indicate individual rats with lines connecting individual between session comparisons. B. These panels show GRAB_DA traces, as described in A, but for cue-primed reinstatement (Cue). Area under the curve for these traces is shown on the Right (0-1 s). Full statistical output for experiments shown in this figure is presented in Tables 3-1 and 3-2. ISOS, isosbestic. Download Figure 3-2, TIF file. [file eneuro-13-ENEURO.0380-25.2026-s006.tif]
