# Supplementary material for: Dopamine and Calcium Dynamics in the Nucleus Accumbens Core during Food Seeking
Source: eNeuro. 2026 Apr 28;13(4):ENEURO.0380-25.2026. doi: 10.1523/ENEURO.0380-25.2026 (PMC13124030; doi:10.1523/ENEURO.0380-25.2026)
Supplement: Table 2-1 — Statistical output for bootstrapping analyses in Figure 2-1 Download Table 2-1, DOCX file. [file eneuro-13-ENEURO.0380-25.2026-s016.docx]

**Table 2-1. Statistical output for bootstrapping analyses in Figure 2-1**

| **Expt phase** | **Measure** | **Factors in analysis** | **Time 95% CI ≠ 0** | **Significantly different?** | **Figure** |
| --- | --- | --- | --- | --- | --- |
| Magazine Training | GCaMP response to magazine entry, z-scored trace (n=11) | Bootstrapping |  | n.s. | 2-1 |
|  |  | MagTrain | n.s. |  |  |
|  |  | SA4 | -0.159 to 6.14 s |  |  |
