# Supplementary material for: Dopamine and Calcium Dynamics in the Nucleus Accumbens Core during Food Seeking
Source: eNeuro. 2026 Apr 28;13(4):ENEURO.0380-25.2026. doi: 10.1523/ENEURO.0380-25.2026 (PMC13124030; doi:10.1523/ENEURO.0380-25.2026)
Supplement: Table 2-2 — Statistical output for bootstrapping analyses in Figure 2-2 Download Table 2-2, DOCX file. [file eneuro-13-ENEURO.0380-25.2026-s017.docx]

**Table 2-2. Statistical output for bootstrapping analyses in Figure 2-2**

| **Expt phase** | **Measure** | **Factors in analysis** | **Time 95% CI ≠ 0** | **Significantly different?** | **Figure** |
| --- | --- | --- | --- | --- | --- |
| SA | GCaMP response to the first lever press, z-scored trace (n=11) | Bootstrapping |  | n.s. | 2-1 A |
|  |  | SA1 | n.s. |  |  |
|  |  | SA4 | n.s. |  |  |
| SA | GCaMP response to the first five lever presses, z-scored trace (n=11) | Bootstrapping |  | 0.311 to 1.62 s | 2-1 B |
|  |  | SA1 | n.s. |  |  |
|  |  | SA4 | 0.477 to 5.17 s |  |  |
| SA | GCaMP response to the lever press in the first 10 min of the session, z-scored trace (n=11) | Bootstrapping |  | 0.241 to 1.53 s | 2-1 C |
|  |  | SA1 | n.s. |  |  |
|  |  | SA4 | 0.449 to 5.20 s |  |  |
| Extinction | GCaMP response to the first lever press, z-scored trace (n=11) | Bootstrapping |  | n.s. | 2-1 D |
|  |  | Ext1 | n.s. |  |  |
|  |  | Ext6 | n.s. |  |  |
| Extinction | GCaMP response to the first five lever presses, z-scored trace (n=11) | Bootstrapping |  | n.s. | 2-1 E |
|  |  | Ext1 | n.s. |  |  |
|  |  | Ext6 | n.s. |  |  |
| Extinction | GCaMP response to the lever press in the first 10 min of the session, z-scored trace (n=11) | Bootstrapping |  | n.s. | 2-1 F |
|  |  | Ext1 | 0.597 to 1.82 s |  |  |
|  |  | Ext6 | n.s. |  |  |
| Extinction/ Reinstatement | GCaMP response to the first lever press, z-scored trace (n=11) | Bootstrapping |  | n.s. | 2-1 G |
|  |  | Ext6 | n.s. |  |  |
|  |  | Cue test | n.s. |  |  |
| Extinction/ Reinstatement | GCaMP response to the first five lever presses, z-scored trace (n=11) | Bootstrapping |  | n.s. | 2-1 H |
|  |  | Ext6 | n.s. |  |  |
|  |  | Cue test | 0.836 to 3.54 s |  |  |
| Extinction/ Reinstatement | GCaMP response to the lever press in the first 10 min of the session, z-scored trace (n=11) | Bootstrapping |  | n.s. | 2-1 I |
|  |  | Ext6 | n.s. |  |  |
|  |  | Cue test | 1.17 to 2.90 s |  |  |
| Extinction/ Reinstatement | GCaMP response to the first lever press, z-scored trace (n=11) | Bootstrapping |  | n.s. | 2-1 J |
|  |  | Ext6 | n.s. |  |  |
|  |  | Pellet+cue test | n.s. |  |  |
| Extinction/ Reinstatement | GCaMP response to the first five lever presses, z-scored trace (n=11) | Bootstrapping |  | n.s. | 2-1 K |
|  |  | Ext6 | n.s. |  |  |
|  |  | Pellet+cue test | n.s. |  |  |
| Extinction/ Reinstatement | GCaMP response to the lever press in the first 10 min of the session, z-scored trace (n=11) | Bootstrapping |  | n.s. | 2-1 L |
|  |  | Ext6 | n.s. |  |  |
|  |  | Pellet+cue test | n.s. |  |  |
