# Supplementary material for: Dopamine and Calcium Dynamics in the Nucleus Accumbens Core during Food Seeking
Source: eNeuro. 2026 Apr 28;13(4):ENEURO.0380-25.2026. doi: 10.1523/ENEURO.0380-25.2026 (PMC13124030; doi:10.1523/ENEURO.0380-25.2026)
Supplement: Table 2-3 — Statistical output for bootstrapping analyses in Figure 2-3 Download Table 2-3, DOCX file. [file eneuro-13-ENEURO.0380-25.2026-s018.docx]

**Table 2-3. Statistical output for bootstrapping analyses in Figure 2-3**

| **Expt phase** | **Measure** | **Factors in analysis** | **Time 95% CI ≠ 0** | **Significantly different?** | **Figure** |
| --- | --- | --- | --- | --- | --- |
| SA | GCaMP response to lever entry, z-scored trace (n=11) | Bootstrapping |  | 1.39 to 5.80 s | 2-3 A, left |
|  |  | SA1 | n.s. |  |  |
|  |  | SA4 | 1.01 to 8.06 s |  |  |
| Extinction | GCaMP response to lever entry, z-scored trace (n=11) | Bootstrapping |  | n.s. | 2-3 B, left |
|  |  | Ext1 | n.s. |  |  |
|  |  | Ext6 | n.s. |  |  |
| Extinction/ Reinstatement | GCaMP response to lever entry, z-scored trace (n=11) | Bootstrapping |  | n.s. | 2-3 C, left |
|  |  | Ext6 | n.s. |  |  |
|  |  | Cue test | n.s. |  |  |
| Extinction/ Reinstatement | GCaMP response to lever entry, z-scored trace (n=11) | Bootstrapping |  | n.s. | 2-3 D, left |
|  |  | Ext6 | n.s. |  |  |
|  |  | Pellet+cue test | n.s. |  |  |
