# Supplementary material for: Dopamine and Calcium Dynamics in the Nucleus Accumbens Core during Food Seeking
Source: eNeuro. 2026 Apr 28;13(4):ENEURO.0380-25.2026. doi: 10.1523/ENEURO.0380-25.2026 (PMC13124030; doi:10.1523/ENEURO.0380-25.2026)
Supplement: Table 2-4 — Statistical output for AUC GCaMP fiber photometry data in Figure 2-3 Download Table 2-4, DOCX file. [file eneuro-13-ENEURO.0380-25.2026-s019.docx]

**Table 2-4. Statistical output for AUC GCaMP fiber photometry data in Figure 2-3**

| **Expt phase** | **Measure** | **Comparison** | **T-value** | **P-value** | **Significant?** | **Figure** |
| --- | --- | --- | --- | --- | --- | --- |
| SA | AUC (n = 11) | SA1 vs. SA4 | t_10_=3.935 | 0.0028 | ** | 3A, right |
| Extinction | AUC (n = 10) | Ext1 vs. Ext6 | t_9_=0.4884 | 0.6370 | n.s. | 3B, right |
| Extinction/ reinstatement | AUC (n = 10) | Ext6 vs. Cue | t_9_=3.382 | 0.0081 | ** | 3C, right |
| Extinction/ reinstatement | AUC (n = 10) | Ext6 vs. Pellet+cue | t_9_=1.509 | 0.01505 | n.s. | 3D, right |
