# Supplementary material for: Dopamine and Calcium Dynamics in the Nucleus Accumbens Core during Food Seeking
Source: eNeuro. 2026 Apr 28;13(4):ENEURO.0380-25.2026. doi: 10.1523/ENEURO.0380-25.2026 (PMC13124030; doi:10.1523/ENEURO.0380-25.2026)
Supplement: Table 3-1 — Statistical output for bootstrapping analyses in Figure 3-2 Download Table 3-1, DOCX file. [file eneuro-13-ENEURO.0380-25.2026-s011.docx]

**Table 3-1. Statistical output for bootstrapping analyses in Figure 3-2**

| **Expt phase** | **Measure** | **Factors in analysis** | **Time 95% CI ≠ 0** | **Significantly different?** | **Figure** |
| --- | --- | --- | --- | --- | --- |
| SA | GRAB_DA response to lever press, z-scored trace (n=11) | Bootstrapping |  | n.s. | 3-2 A, left |
|  |  | With 405 nm | -4.9 to -1.43 s, 1.16 to 0.490 s, 0.726 to 1.67 s, 1.18 to 10 s |  |  |
|  |  | Without 405 nm | -4.9 to -1.43 s, 1.16 to 0.490 s, 1.18 to 10 s |  |  |
| Reinstatement | GRAB_DA response to lever press, z-scored trace (n=11) | Bootstrapping |  | n.s. | 3-2 B, left |
|  |  | With 405 nm | n.s. |  |  |
|  |  | Without 405 nm | n.s. |  |  |
