# Supplementary material for: Dopamine and Calcium Dynamics in the Nucleus Accumbens Core during Food Seeking
Source: eNeuro. 2026 Apr 28;13(4):ENEURO.0380-25.2026. doi: 10.1523/ENEURO.0380-25.2026 (PMC13124030; doi:10.1523/ENEURO.0380-25.2026)
Supplement: Table 3-2 — Statistical output for AUC GRAB_DA fiber photometry data in Figure 3-2. Download Table 3-2, DOCX file. [file eneuro-13-ENEURO.0380-25.2026-s012.docx]

**Table 3-2. Statistical output for AUC GRAB_DA fiber photometry data in Figure 3-2**

| **Expt phase** | **Measure** | **Comparison** | **T-value** | **P-value** | **Significant?** | **Figure** |
| --- | --- | --- | --- | --- | --- | --- |
| SA | AUC (n = 11) | With 405 nm vs. Without 405 nm | t_10_=0.1275 | 0.9011 | n.s. | 3-2 A, right |
| Reinstatement | AUC (n = 11) | With 405 nm vs. Without 405 nm | t_10_=0.8460 | 0.4173 | n.s. | 3-2 B, right |
