# Supplementary material for: Dopamine and Calcium Dynamics in the Nucleus Accumbens Core during Food Seeking
Source: eNeuro. 2026 Apr 28;13(4):ENEURO.0380-25.2026. doi: 10.1523/ENEURO.0380-25.2026 (PMC13124030; doi:10.1523/ENEURO.0380-25.2026)
Supplement: Table 3-3 — Statistical output for bootstrapping analyses in Figure 3-3 Download Table 3-3, DOCX file. [file eneuro-13-ENEURO.0380-25.2026-s013.docx]

**Table 3-3. Statistical output for bootstrapping analyses in Figure 3-3**

| **Expt phase** | **Measure** | **Factors in analysis** | **Time 95% CI ≠ 0** | **Significantly different?** | **Figure** |
| --- | --- | --- | --- | --- | --- |
| SA | GRAB_DA response to the first lever press, z-scored trace (n=11) | Bootstrapping |  | n.s. | 3-2 A |
|  |  | SA1 | n.s. |  |  |
|  |  | SA4 | 3.87 to 4.92 s |  |  |
| SA | GRAB_DA response to the first 5 lever presses, z-scored trace (n=11) | Bootstrapping |  | 2.75 to 6.06 s | 3-2 B |
|  |  | SA1 | 2.16 to 4.40 s |  |  |
|  |  | SA4 | -4.40 to -3.26 s, 2.93 to 7.85 s |  |  |
| SA | GRAB_DA response to the lever press in the first 10 min, z-scored trace (n=11) | Bootstrapping |  | -2.27 to -1.21 s, 3.54 to 4.95 s | 3-2 C |
|  |  | SA1 | n.s. |  |  |
|  |  | SA4 | -5 to -1.79 s, -0.655 to 0.393 s, 3.49 to 6.10 s |  |  |
| Extinction | GRAB_DA response to the first lever press, z-scored trace (n=11) | Bootstrapping |  | n.s. | 3-2 D |
|  |  | Ext1 | n.s. |  |  |
|  |  | Ext6 | n.s. |  |  |
| Extinction | GRAB_DA response to the first 5 lever presses, z-scored trace (n=11) | Bootstrapping |  | n.s. | 3-2 E |
|  |  | Ext1 | 4.41 to 5.66 s, 7.77 to 9.26 s |  |  |
|  |  | Ext6 | n.s. |  |  |
| Extinction | GRAB_DA response to the lever press in the first 10 min, z-scored trace (n=11) | Bootstrapping |  | n.s. | 3-2 F |
|  |  | Ext1 | 1.76 to 9.98 s |  |  |
|  |  | Ext6 | n.s. |  |  |
| Extinction/ Reinstatement | GRAB_DA response to the first lever press, z-scored trace (n=11) | Bootstrapping |  | n.s. | 3-2 G |
|  |  | Ext6 | n.s. |  |  |
|  |  | Cue test | n.s. |  |  |
| Extinction/ Reinstatement | GRAB_DA response to the first 5 lever presses, z-scored trace (n=11) | Bootstrapping |  | n.s. | 3-2 H |
|  |  | Ext6 | n.s. |  |  |
|  |  | Cue test | 0.131 to 4.45 s |  |  |
| Extinction/ Reinstatement | GRAB_DA response to the lever press in the first 10, z-scored trace (n=11) | Bootstrapping |  | n.s. | 3-2 I |
|  |  | Ext6 | n.s. |  |  |
|  |  | Cue test | n.s. |  |  |
| Extinction/ Reinstatement | GRAB_DA response to the first lever press, z-scored trace (n=11) | Bootstrapping |  | n.s. | 3-2 J |
|  |  | Ext6 | n.s. |  |  |
|  |  | Pellet+cue test | n.s. |  |  |
| Extinction/ Reinstatement | GRAB_DA response to the first 5 lever presses, z-scored trace (n=11) | Bootstrapping |  | n.s. | 3-2 K |
|  |  | Ext6 | n.s. |  |  |
|  |  | Pellet+cue test | n.s. |  |  |
| Extinction/ Reinstatement | GRAB_DA response to the lever press in the first 10 min, z-scored trace (n=11) | Bootstrapping |  | n.s. | 3-2 L |
|  |  | Ext6 | n.s. |  |  |
|  |  | Pellet+cue test | n.s. |  |  |
