# Supplementary material for: Dopamine and Calcium Dynamics in the Nucleus Accumbens Core during Food Seeking
Source: eNeuro. 2026 Apr 28;13(4):ENEURO.0380-25.2026. doi: 10.1523/ENEURO.0380-25.2026 (PMC13124030; doi:10.1523/ENEURO.0380-25.2026)
Supplement: Table 3-4 — Statistical output for bootstrapping analyses in Figure 3-4 Download Table 3-4, DOCX file. [file eneuro-13-ENEURO.0380-25.2026-s014.docx]

**Table 3-4. Statistical output for bootstrapping analyses in Figure 3-4**

| **Expt phase** | **Measure** | **Factors in analysis** | **Time 95% CI ≠ 0** | **Significantly different?** | **Figure** |
| --- | --- | --- | --- | --- | --- |
| SA | GRAB_DA response to lever entry, z-scored trace (n=11) | Bootstrapping |  | 3.54 to 9.98 s | 3-4 A, left |
|  |  | SA1 | 0.891 to 2.22 s, 3.41 to 7.66 s |  |  |
|  |  | SA4 | 0.145 to 2.54 s, 4.81 to 7.27 s, 7.44 to 9.06 s |  |  |
| Extinction | GRAB_DA response to lever entry, z-scored trace (n=11) | Bootstrapping |  | n.s. | 3-4 B, left |
|  |  | Ext1 | n.s. |  |  |
|  |  | Ext6 | n.s. |  |  |
| Extinction/ Reinstatement | GRAB_DA response to lever entry, z-scored trace (n=11) | Bootstrapping |  | n.s. | 3-4 C, left |
|  |  | Ext6 | n.s. |  |  |
|  |  | Cue test | n.s. |  |  |
| Extinction/ Reinstatement | GRAB_DA response to lever entry, z-scored trace (n=11) | Bootstrapping |  | n.s. | 3-4 D, left |
|  |  | Ext6 | n.s. |  |  |
|  |  | Pellet+cue test | n.s. |  |  |
